# Supplementary material for: Implementation strategies to improve outcomes in patients with established cardiovascular disease in sub-Saharan Africa: A systematic review
Source: PLOS Glob Public Health. 2025 May 5;5(5):e0004544. doi: 10.1371/journal.pgph.0004544 (PMC12052185; doi:10.1371/journal.pgph.0004544)
Supplement: S1 Text — (DOCX) [file pgph.0004544.s003.docx]

S1 Text

Legend: The text below details the search criteria stratified by database

GLOBAL HEALTH SEARCH

| **Query** | **Results from 8 Jan 2024** | hits |
| --- | --- | --- |
| 1 | Patient Care Management.mp. | 17 |
| 2 | Comprehensive Health Care.mp. | 232 |
| 3 | Nursing Process.mp. | 90 |
| 4 | 'Nursing Assessment'.mp. | 43 |
| 5 | Patient Care Planning.mp. or planning.sh. or health care.sh. | 172,507 |
| 6 | Case Management.mp. | 3,521 |
| 7 | delivery of health care.mp. or primary health care.sh. | 25,782 |
| 8 | Delivery of Health Care, Integrated.mp. or health services.sh. | 125,672 |
| 9 | 'Managed Care Programs'.mp. | 7 |
| 10 | Disease Management.mp. | 3,736 |
| 11 | 'Patient Care Team'.mp. or physicians.sh. or Community care.sh. or nursing.sh. or general practitioners.sh. or patient care.sh. | 60,100 |
| 12 | 'Primary Health Care'.mp. or health care workers.sh. or health care.sh. or primary health care.sh. | 199,675 |
| 13 | Reminder Systems.mp. | 173 |
| 14 | Guideline Adherence.mp. | 384 |
| 15 | Home Care Services.mp. or medical services.sh. or health services.sh. | 130,409 |
| 16 | Home Nursing.mp. | 175 |
| 17 | 'Nursing Services'.mp. or nursing.sh. or nurses.sh. or medical services.sh. | 32,203 |
| 18 | 'Professional Role'.mp. or health care workers.sh. or medical auxiliaries.sh. | 56,549 |
| 19 | Community Health Services.mp. or community health services/ | 6,260 |
| 20 | Medical Records/ or Medical Records Systems, Computerized.mp. [mp=abstract, title, original title, heading words, cabicodes words] | 5,584 |
| 21 | 'Patient Compliance'.mp. or patient compliance.sh. | 7,846 |
| 22 | Life Style.mp. or food intake.sh. or exercise.sh. or lifestyle.sh. or physical activity.sh. | 158,611 |
| 23 | Health Promotion.mp. or health promotion/ | 39,466 |
| 24 | Health Services Administration.mp. or health services.sh. | 125,707 |
| 25 | Education, Medical, Continuing.mp. or continuing education.sh. or medical education.sh. or education.sh. | 65,286 |
| 26 | Marketing of Health Services.mp. | 5 |
| 27 | Patient Participation.mp. or patient compliance.sh. or participation.sh. | 13,483 |
| 28 | Quality of Health Care.mp. or "quality of care".sh. | 12,551 |
| 29 | Quality Assurance, Health Care.mp. | 1 |
| 30 | Exercise/ or Physical Fitness.mp. [mp=abstract, title, original title, heading words, cabicodes words] | 46,497 |
| 31 | Smoking Cessation.mp. or smoking cessation/ | 11,879 |
| 32 | Diet/ or Fat-restricted/ or Carbohydrate-Restricted.mp. [mp=abstract, title, original title, heading words, cabicodes words] | 74,545 |
| 33 | (Alcohol Drinking prevention or control).mp. [mp=abstract, title, original title, heading words, cabicodes words] | 1,128,244 |
| 34 | Health Education.mp. or health education/ | 35,450 |
| 35 | Community Health Planning.mp. or community health.sh. or planning.sh. | 28,881 |
| 36 | Communication/ or Communication Barriers/ or Information Dissemination/ or Interdisciplinary Communication.mp. [mp=abstract, title, original title, heading words, cabicodes words] | 11,097 |
| 37 | Nurse Clinicians.mp. | 11 |
| 38 | Nurse Practitioners.mp. or nurses.sh. | 14,089 |
| 39 | Risk Reduction Behavior.mp. or risk reduction.sh. | 22,200 |
| 40 | Pamphlets.mp. | 1,036 |
| 41 | Health Behavior.mp. or behaviour.sh. or health behaviour.sh. | 107,612 |
| 42 | Health Knowledge, Attitudes, Practice.mp. or attitudes.sh. or health education.sh. | 104,341 |
| 43 | Secondary Prevention.mp. or prevention.sh. | 34,745 |
| 44 | Preventive Health Services.mp. or preventive medicine.sh. | 6,412 |
| 45 | (manag$ adj3 care).tw. | 6,218 |
| 46 | (management adj3 program$).tw. | 5,345 |
| 47 | (case adj3 manag$).tw. | 5,078 |
| 48 | (patient adj3 management).tw. | 4,470 |
| 49 | (home adj3 intervention$).tw. | 1,004 |
| 50 | (home adj visit$).tw. | 3,568 |
| 51 | (discharg$ adj3 program$).tw. | 161 |
| 52 | (practice adj guideline$).tw. | 3,472 |
| 53 | (discharg$ adj3 plan$).tw. | 709 |
| 54 | (comprehensive adj3 care).tw. | 2,539 |
| 55 | (treatment adj3 plan$).tw. | 26,407 |
| 56 | (nurse$ adj3 led).tw. | 564 |
| 57 | (disease adj management).tw. | 3,736 |
| 58 | multidisciplin$.tw. | 13,055 |
| 59 | secondary prevention clinic$.tw. | 6 |
| 60 | reminder$.tw. | 3,651 |
| 61 | recall$.tw. | 23,082 |
| 62 | (nurse adj3 clinic$).tw. | 413 |
| 63 | (secondary prevention adj3 intervention$).tw. | 107 |
| 64 | (secondary prevention adj3 program$).tw. | 148 |
| 65 | Appointments.mp. and Schedules/ [mp=abstract, title, original title, heading words, cabicodes words] | 0 |
| 66 | appointment$.tw. | 5,829 |
| 67 | (outreach adj nurs$).tw. | 11 |
| 68 | (outreach adj visit$).tw. | 84 |
| 69 | (lifestyle adj3 intervention$).tw. | 4,691 |
| 70 | (nurs$ adj intervention$).tw. | 649 |
| 71 | (education$ adj program$).tw. | 16,143 |
| 72 | (physical adj (activit$ or exercise$)).tw. | 80,579 |
| 73 | (exercise adj3 (train$ or intervention$ or program$ or activit$ or regim$)).tw. | 11,370 |
| 74 | fitness.tw. | 21,123 |
| 75 | (risk factor$ adj3 (modif$ or reduc$ or manage$ or monitor$ or self manage$)).tw. | 9,485 |
| 76 | (guideline directed medical therap* or GDMT).tw. | 25 |
| 77 | (guideline directed medical therap* or GDMT).mp. | 25 |
| 78 | (medic* adj3 uptit*).mp. | 3 |
| 79 | "Africa South of the Sahara".mp. or "Africa South of Sahara"/ | 268,733 |
| 80 | ("Africa South of the Sahara" or sub-Saharan Africa or subSaharan Africa).ti,ab. | 20,602 |
| 81 | Central Africa.tw. | 26,214 |
| 82 | Eastern Africa.tw. | 760 |
| 83 | Southern Africa.tw. | 64,686 |
| 84 | Western Africa.tw. | 608 |
| 85 | Angola.mp. or Angola/ | 2,018 |
| 86 | Angola.ti,ab. | 1,805 |
| 87 | Cameroon.mp. or Cameroon/ | 9,645 |
| 88 | (Cameroon or Kamerun or Cameroun).ti,ab. | 8,046 |
| 89 | Cape Verde.mp. or Cape Verde/ | 447 |
| 90 | (Cape Verde or Cabo Verde).ti,ab. | 436 |
| 91 | Comoros.mp. or Comoros/ | 465 |
| 92 | (Comoros or Glorioso Islands or Mayotte).ti,ab. | 566 |
| 93 | Congo.mp. or Congo Basin/ or Congo/ or Congo River/ or Congo Democratic Republic/ | 15,348 |
| 94 | (Congo not ((Democratic Republic adj3 Congo) or congo red or crimean-congo)).ti,ab. | 5,333 |
| 95 | Cote d'Ivoire.mp. or Cote d'Ivoire/ | 5,763 |
| 96 | (Cote d'Ivoire or Cote dIvoire or Ivory Coast).ti,ab. | 5,333 |
| 97 | Eswatini.mp. or Eswatini/ | 1,160 |
| 98 | (eSwatini or Swaziland).ti,ab. | 1,109 |
| 99 | Ghana.mp. or Ghana/ | 14,960 |
| 100 | (Ghana or Gold Coast).ti,ab. | 13,445 |
| 101 | Kenya.mp. or Kenya/ | 23,690 |
| 102 | (Kenya or East Africa Protectorate).ti,ab. | 20,550 |
| 103 | Lesotho.mp. or Lesotho/ | 946 |
| 104 | (Lesotho or Basutoland).ti,ab. | 821 |
| 105 | Mauritania.mp. or Mauritania/ | 812 |
| 106 | Mauritania.ti,ab. | 709 |
| 107 | Nigeria.mp. or Nigeria/ | 44,957 |
| 108 | Nigeria.ti,ab. | 37,515 |
| 109 | (Sao Tome adj2 Principe).ti,ab. | 161 |
| 110 | Senegal/ or Senegal.mp. | 8,277 |
| 111 | Senegal.ti,ab. | 6,447 |
| 112 | Sudan.mp. or Southern Sudan/ or South Sudan/ or Sudan/ | 10,460 |
| 113 | (Sudan not South Sudan).ti,ab. | 8,316 |
| 114 | Zambia.mp. or Zambia/ | 7,027 |
| 115 | (Zambia or Northern Rhodesia).ti,ab. | 6,029 |
| 116 | Zimbabwe.mp. or Zimbabwe/ | 8,182 |
| 117 | (Zimbabwe or Southern Rhodesia).ti,ab. | 5,851 |
| 118 | Botswana.mp. or Botswana/ | 2,626 |
| 119 | (Botswana or Bechuanaland or Kalahari).ti,ab. | 2,466 |
| 120 | Equatorial Guinea.mp. or Equatorial Guinea/ | 611 |
| 121 | (Equatorial Guinea or Spanish Guinea).ti,ab. | 514 |
| 122 | Gabon.mp. or Gabon/ | 2,225 |
| 123 | (Gabon or Gabonese Republic).ti,ab. | 1,899 |
| 124 | Mauritius.mp. or Mauritius/ | 1,217 |
| 125 | (Mauritius or Agalega Islands).ti,ab. | 1,072 |
| 126 | Namibia.mp. or Namibia/ | 1,571 |
| 127 | (Namibia or German South West Africa).ti,ab. | 1,215 |
| 128 | South Africa.mp. or South Africa/ | 42,987 |
| 129 | (South Africa or Cape Colony or British Bechuanaland or Boer Republics or Zululand or Transvaal or Natalia Republic or Orange Free State).ti,ab. | 30,860 |
| 130 | Benin.mp. or Benin/ | 4,722 |
| 131 | (Benin or Dahomey).ti,ab. | 4,359 |
| 132 | Burkina Faso.mp. or Burkina Faso/ | 6,441 |
| 133 | (Burkina Faso or Burkina Fasso or Upper Volta).ti,ab. | 6,002 |
| 134 | Burundi.mp. or Burundi/ | 1,113 |
| 135 | (Burundi or Ruanda-Urundi).ti,ab. | 1,206 |
| 136 | Central African Republic/ | 1,260 |
| 137 | (Central African Republic or Ubangi-Shari).ti,ab. | 1,267 |
| 138 | Chad/ | 1,549 |
| 139 | Chad.ti,ab. | 1,580 |
| 140 | Democratic Republic of the Congo/ | 8,556 |
| 141 | (((Democratic Republic or DR) adj2 Congo) or Congo-Kinshasa or Belgian Congo or Zaire or Congo Free State).ti,ab. | 6,904 |
| 142 | Eritrea/ | 833 |
| 143 | Eritrea.ti,ab. | 773 |
| 144 | Ethiopia/ | 21,743 |
| 145 | (Ethiopia or Abyssinia).ti,ab. | 20,603 |
| 146 | Gambia/ | 3,410 |
| 147 | Gambia.ti,ab. | 2,939 |
| 148 | Guinea/ | 1,660 |
| 149 | (Guinea not (New Guinea or Guinea Pig* or Guinea Fowl or Guinea-Bissau or Portuguese Guinea or Equatorial Guinea)).ti,ab. | 3,422 |
| 150 | Guinea-Bissau/ | 1,158 |
| 151 | (Guinea-Bissau or Portuguese Guinea).ti,ab. | 1,128 |
| 152 | (Guinea-Bissau or Portuguese Guinea).ti,ab. | 1,128 |
| 153 | Liberia/ | 2,027 |
| 154 | Liberia.ti,ab. | 2,036 |
| 155 | Madagascar/ | 4,433 |
| 156 | (Madagascar or Malagasy Republic).ti,ab. | 4,386 |
| 157 | Malawi/ | 8,386 |
| 158 | (Malawi or Nyasaland).ti,ab. | 7,715 |
| 159 | Mali/ | 3,914 |
| 160 | Mali.ti,ab. | 3,766 |
| 161 | Mozambique/ | 4,143 |
| 162 | (Mozambique or Mocambique or Portuguese East Africa).ti,ab. | 4,027 |
| 163 | Niger/ | 1,723 |
| 164 | (Niger not (Aspergillus or Peptococcus or Schizothorax or Cruciferae or Gobius or Lasius or Agelastes or Melanosuchus or radish or Parastromateus or Orius or Apergillus or Parastromateus or Stomoxys)).ti,ab. | 4,237 |
| 165 | Rwanda/ | 3,308 |
| 166 | (Rwanda or Ruanda).ti,ab. | 3,188 |
| 167 | Sierra Leone/ | 2,874 |
| 168 | (Sierra Leone or Salone).ti,ab. | 2,820 |
| 169 | Somalia/ | 2,212 |
| 170 | (Somalia or Somaliland).ti,ab. | 1,893 |
| 171 | South Sudan/ | 634 |
| 172 | South Sudan.ti,ab. | 740 |
| 173 | Tanzania/ | 16,791 |
| 174 | (Tanzania or Tanganyika or Zanzibar).ti,ab. | 15,920 |
| 175 | Togo/ | 2,087 |
| 176 | (Togo or Togolese Republic or Togoland).ti,ab. | 1,905 |
| 177 | Uganda/ | 17,524 |
| 178 | Uganda.ti,ab. | 16,518 |
| 179 | Seychelles/ | 421 |
| 180 | Seychelles.ti,ab. | 422 |
| 181 | or/79-180 [ALL SUB-SAHARAN AFRICA COUNTRIES] | 289,036 |
| 182 | cardiovascular diseases.sh. | 69,616 |
| 183 | cardio*.tw. | 153,011 |
| 184 | cardia*.tw. | 52,067 |
| 185 | heart*.tw. | 144,945 |
| 186 | coronary*.tw. | 71,200 |
| 187 | angina*.tw. | 3,961 |
| 188 | ventric*.tw. | 18,566 |
| 189 | myocard*.tw. | 39,180 |
| 190 | pericard*.tw. | 4,066 |
| 191 | isch?em*.tw. | 30,174 |
| 192 | emboli*.tw. | 6,301 |
| 193 | arrhythmi*.tw. | 6,141 |
| 194 | thrombo*.tw. | 39,918 |
| 195 | atrial fibrillat*.tw. | 3,616 |
| 196 | tachycardi*.tw. | 3,627 |
| 197 | endocardi*.tw. | 9,475 |
| 198 | (sick adj sinus).tw. | 59 |
| 199 | 'Stroke'/ | 16,845 |
| 200 | (stroke or stokes).tw. | 28,183 |
| 201 | cerebrovasc*.tw. | 12,041 |
| 202 | cerebral vascular.tw. | 467 |
| 203 | apoplexy.tw. | 196 |
| 204 | (brain adj2 accident*).tw. | 12 |
| 205 | ((brain* or cerebral or lacunar) adj2 infarct*).tw. | 3,239 |
| 206 | 'Hyperlipidemias'.mp. | 115 |
| 207 | hyperlipid*.tw. | 11,326 |
| 208 | hyperlip?emia*.tw. | 12,406 |
| 209 | hypercholesterol*.tw. | 13,098 |
| 210 | hypercholester?emia*.tw. | 204 |
| 211 | hyperlipoprotein?emia*.tw. | 1,165 |
| 212 | hypertriglycerid?emia*.tw. | 5,912 |
| 213 | 'Arteriosclerosis'.mp. | 22,257 |
| 214 | cholesterol/ or 'Cholesterol'.mp. | 109,109 |
| 215 | cholesterol.tw. | 109,109 |
| 216 | or/182-215 | 426,629 |
| 217 | or/1-78 | 1,775,836 |
| 218 | 181 and 216 and 217 | 5,310 |

EMBASE SEARCH

| **Query** | **Results from 8 Jan 2024** |  |
| --- | --- | --- |
| 1 | Patient Care Management/ | 343,543 |
| 2 | Comprehensive Health Care/ | 136,567 |
| 3 | Nursing Process/ | 7,181 |
| 4 | 'nursing assessment'/ | 27,582 |
| 5 | Patient Care Planning/ | 31,171 |
| 6 | Case Management/ | 13,849 |
| 7 | delivery of health care/ | 177,915 |
| 8 | Delivery of Health Care, Integrated/ | 12,206 |
| 9 | Managed Care Programs'/ | 149,774 |
| 10 | Disease Management/ | 25,166 |
| 11 | Patient Care Team'/ | 376 |
| 12 | Primary Health Care'/ | 80,832 |
| 13 | Reminder Systems/ | 2,930 |
| 14 | Guideline Adherence/ | 18,628 |
| 15 | Home Care Services/ | 62,959 |
| 16 | Home Nursing/ | 71,590 |
| 17 | 'Nursing Services'/ | 232,814 |
| 18 | 'Professional Role'/ | 37,080 |
| 19 | Community Health Services/ | 58,630 |
| 20 | Medical Records/ or Medical Records Systems, Computerized/ | 195,311 |
| 21 | Patient Compliance'/ | 155,803 |
| 22 | Life Style/ | 127,087 |
| 23 | Health Promotion/ | 113,237 |
| 24 | Health Services Administration/ | 182,986 |
| 25 | Education, Medical, Continuing/ | 254,245 |
| 26 | Marketing of Health Services/ | 121,705 |
| 27 | Patient Participation/ | 36,663 |
| 28 | Quality of Health Care/ | 237,273 |
| 29 | Quality Assurance, Health Care/ | 224,539 |
| 30 | Exercise/ or Physical Fitness/ | 408,815 |
| 31 | Smoking Cessation/ | 70,405 |
| 32 | Diet/ or Fat-restricted/ or Carbohydrate-Restricted/ | 316,655 |
| 33 | Alcohol Drinking/pc [Prevention & Control] | 1,060 |
| 34 | Health Education/ | 117,433 |
| 35 | Community Health Planning/ | 115,717 |
| 36 | Communication/ or Communication Barriers/ or Information Dissemination/ or Interdisciplinary Communication/ | 194,877 |
| 37 | Nurse Clinicians/ | 2,887 |
| 38 | Nurse Practitioners/ | 25,667 |
| 39 | Risk Reduction Behavior/ | 126,495 |
| 40 | Pamphlets/ | 195,947 |
| 41 | Health Behavior/ | 81,616 |
| 42 | Health Knowledge, Attitudes, Practice/ | 112,236 |
| 43 | Secondary Prevention/ | 36,003 |
| 44 | Preventive Health Services/ | 32,621 |
| 45 | (manag$ adj3 care).ab,ti,kw. | 81,117 |
| 46 | (management adj3 program$).ab,ti,kw. | 33,111 |
| 47 | (case adj3 manag$).ab,ti,kw. | 37,262 |
| 48 | (patient adj3 management).ab,ti,kw. | 75,293 |
| 49 | (home adj3 intervention$).ab,ti,kw. | 5,874 |
| 50 | (home adj visit$).ab,ti,kw. | 14,800 |
| 51 | (discharg$ adj3 program$).ab,ti,kw. | 2,376 |
| 52 | (practice adj guideline$).ab,ti,kw. | 47,610 |
| 53 | (discharg$ adj3 plan$).ab,ti,kw. | 10,009 |
| 54 | (comprehensive adj3 care).ab,ti,kw. | 20,384 |
| 55 | (treatment adj3 plan$).ab,ti,kw. | 151,290 |
| 56 | (nurse$ adj3 led).ab,ti,kw. | 10,146 |
| 57 | (disease adj management).ab,ti,kw. | 31,367 |
| 58 | multidisciplin$.ab,ti,kw. | 201,059 |
| 59 | secondary prevention clinic$.ab,ti,kw. | 122 |
| 60 | reminder$.ab,ti,kw. | 26,523 |
| 61 | recall$.ab,ti,kw. | 123,395 |
| 62 | (nurse adj3 clinic$).ab,ti,kw. | 12,232 |
| 63 | (secondary prevention adj3 intervention$).ab,ti,kw. | 810 |
| 64 | (secondary prevention adj3 program$).ab,ti,kw. | 1,194 |
| 65 | Appointments.mp. and Schedules/ [mp=title, abstract, heading word, drug trade name, original title, device manufacturer, drug manufacturer, device trade name, keyword heading word, floating subheading word, candidate term word] | 0 |
| 66 | appointment$.ab,ti,kw. | 62,130 |
| 67 | (outreach adj nurs$).ab,ti,kw. | 215 |
| 68 | (outreach adj visit$).ab,ti,kw. | 445 |
| 69 | (lifestyle adj3 intervention$).ab,ti,kw. | 17,833 |
| 70 | (nurs$ adj intervention$).ab,ti,kw. | 10,866 |
| 71 | (education$ adj program$).ab,ti,kw. | 71,087 |
| 72 | (physical adj (activit$ or exercise$)).tw. | 233,523 |
| 73 | (exercise adj3 (train$ or intervention$ or program$ or activit$ or regim$)).ab,ti,kw. | 88,505 |
| 74 | fitness.ab,ti,kw. | 114,914 |
| 75 | (risk factor$ adj3 (modif$ or reduc$ or manage$ or monitor$ or self manage$)).ab,ti,kw. | 50,279 |
| 76 | (guideline directed medical therap* or GDMT).ab,ti,kw. | 2,530 |
| 77 | (guideline directed medical therap* or GDMT).mp. | 2,582 |
| 78 | (medic* adj3 uptit*).mp. | 80 |
| 79 | Africa South of the Sahara'/ | 18,412 |
| 80 | ("Africa South of the Sahara" or sub-Saharan Africa or subSaharan Africa).ab,ti. | 35,346 |
| 81 | Central Africa.ab,ti,kw. | 4,629 |
| 82 | Eastern Africa.ab,ti,kw. | 1,522 |
| 83 | Southern Africa.ab,ti,kw. | 6,175 |
| 84 | Western Africa.ab,ti,kw. | 1,153 |
| 85 | Angola/ | 1,896 |
| 86 | Angola.ab,ti. | 2,067 |
| 87 | Cameroon/ | 9,277 |
| 88 | (Cameroon or Kamerun or Cameroun).ab,ti. | 10,436 |
| 89 | Cape Verde/ | 554 |
| 90 | (Cape Verde or Cabo Verde).ab,ti. | 832 |
| 91 | Comoros/ | 443 |
| 92 | (Comoros or Glorioso Islands or Mayotte).ab,ti. | 775 |
| 93 | Congo/ | 4,794 |
| 94 | (Congo not ((Democratic Republic adj3 Congo) or congo red or crimean-congo)).ab,ti. | 4,028 |
| 95 | Cote d'Ivoire/ | 3,998 |
| 96 | (Cote d'Ivoire or Cote dIvoire or Ivory Coast).ab,ti. | 5,433 |
| 97 | Eswatini/ | 357 |
| 98 | (eSwatini or Swaziland).ab,ti. | 1,413 |
| 99 | Ghana/ | 16,865 |
| 100 | (Ghana or Gold Coast).ab,ti. | 18,393 |
| 101 | Kenya/ | 27,636 |
| 102 | (Kenya or East Africa Protectorate).ab,ti. | 27,697 |
| 103 | Lesotho/ | 1,038 |
| 104 | (Lesotho or Basutoland).ab,ti. | 1,129 |
| 105 | Mauritania/ | 827 |
| 106 | Mauritania.ab,ti. | 866 |
| 107 | Nigeria/ | 48,843 |
| 108 | Nigeria.ab,ti. | 47,349 |
| 109 | (Sao Tome adj2 Principe).ab,ti. | 224 |
| 110 | Senegal/ | 8,238 |
| 111 | Senegal.ab,ti. | 8,259 |
| 112 | Sudan/ | 8,795 |
| 113 | (Sudan not South Sudan).ab,ti. | 11,937 |
| 114 | Zambia/ | 8,321 |
| 115 | (Zambia or Northern Rhodesia).ab,ti. | 8,211 |
| 116 | Zimbabwe/ | 8,532 |
| 117 | (Zimbabwe or Southern Rhodesia).ab,ti. | 7,580 |
| 118 | Botswana/ | 3,498 |
| 119 | (Botswana or Bechuanaland or Kalahari).ab,ti. | 3,915 |
| 120 | Equatorial Guinea/ | 600 |
| 121 | (Equatorial Guinea or Spanish Guinea).ab,ti. | 700 |
| 122 | Gabon/ | 2,085 |
| 123 | (Gabon or Gabonese Republic).ab,ti. | 2,354 |
| 124 | Mauritius/ | 1,122 |
| 125 | (Mauritius or Agalega Islands).ab,ti. | 1,301 |
| 126 | Namibia/ | 2,136 |
| 127 | (Namibia or German South West Africa).ab,ti. | 2,280 |
| 128 | South Africa/ | 64,436 |
| 129 | (South Africa or Cape Colony or British Bechuanaland or Boer Republics or Zululand or Transvaal or Natalia Republic or Orange Free State).ti,ab. | 54,478 |
| 130 | Benin/ | 3,324 |
| 131 | (Benin or Dahomey).ab,ti. | 5,794 |
| 132 | Burkina Faso/ | 5,679 |
| 133 | (Burkina Faso or Burkina Fasso or Upper Volta).ab,ti. | 6,497 |
| 134 | Burundi/ | 1,139 |
| 135 | (Burundi or Ruanda-Urundi).ab,ti. | 1,282 |
| 136 | Central African Republic/ | 1,109 |
| 137 | (Central African Republic or Ubangi-Shari).ab,ti. | 1,325 |
| 138 | Chad/ | 1,186 |
| 139 | Chad.ab,ti. | 1,972 |
| 140 | Democratic Republic of the Congo/ | 5,344 |
| 141 | (((Democratic Republic or DR) adj2 Congo) or Congo-Kinshasa or Belgian Congo or Zaire or Congo Free State).ab,ti. | 6,568 |
| 142 | Eritrea/ | 762 |
| 143 | Eritrea.ti,ab. | 886 |
| 144 | Ethiopia/ | 28,934 |
| 145 | (Ethiopia or Abyssinia).ti,ab. | 29,466 |
| 146 | Gambia/ | 3,412 |
| 147 | Gambia.ti,ab. | 3,203 |
| 148 | Guinea/ | 3,373 |
| 149 | (Guinea not (New Guinea or Guinea Pig* or Guinea Fowl or Guinea-Bissau or Portuguese Guinea or Equatorial Guinea)).ab,ti. | 3,993 |
| 150 | Guinea-Bissau/ | 1,283 |
| 151 | (Guinea-Bissau or Portuguese Guinea).ab,ti. | 1,345 |
| 152 | Liberia/ | 2,235 |
| 153 | Liberia.ti,ab. | 2,284 |
| 154 | Madagascar/ | 5,563 |
| 155 | (Madagascar or Malagasy Republic).ab,ti. | 6,499 |
| 156 | Malawi/ | 10,045 |
| 157 | (Malawi or Nyasaland).ab,ti. | 10,764 |
| 158 | Mali/ | 4,368 |
| 159 | Mali.ti,ab. | 5,482 |
| 160 | Mozambique/ | 5,043 |
| 161 | (Mozambique or Mocambique or Portuguese East Africa).ab,ti. | 5,515 |
| 162 | Niger/ | 2,996 |
| 163 | (Niger not (Aspergillus or Peptococcus or Schizothorax or Cruciferae or Gobius or Lasius or Agelastes or Melanosuchus or radish or Parastromateus or Orius or Apergillus or Parastromateus or Stomoxys)).ti,ab. | 5,239 |
| 164 | Rwanda/ | 5,089 |
| 165 | (Rwanda or Ruanda).ab,ti. | 5,175 |
| 166 | Sierra Leone/ | 3,191 |
| 167 | (Sierra Leone or Salone).ab,ti. | 3,428 |
| 168 | Somalia/ | 2,577 |
| 169 | (Somalia or Somaliland).ab,ti. | 2,256 |
| 170 | South Sudan/ | 562 |
| 171 | South Sudan.ab,ti. | 958 |
| 172 | Tanzania/ | 20,287 |
| 173 | (Tanzania or Tanganyika or Zanzibar).ab,ti. | 20,999 |
| 174 | Togo/ | 1,865 |
| 175 | (Togo or Togolese Republic or Togoland).ab,ti. | 2,139 |
| 176 | Uganda/ | 24,333 |
| 177 | Uganda.ab,ti. | 23,892 |
| 178 | Seychelles/ | 584 |
| 179 | Seychelles.ab,ti. | 930 |
| 180 | or/79-179 [ALL SUB-SAHARAN AFRICA COUNTRIES] | 413,514 |
| 181 | Cardiovascular Diseases'/ | 124,215 |
| 182 | cardio*.ab,ti,kw. | 1,526,046 |
| 183 | cardia*.ab,ti,kw. | 1,154,573 |
| 184 | heart*.ab,ti,kw. | 1,534,626 |
| 185 | coronary*.ab,ti,kw. | 697,481 |
| 186 | angina*.ab,ti,kw. | 92,422 |
| 187 | ventric*.ab,ti,kw. | 738,078 |
| 188 | myocard*.ab,ti,kw. | 679,750 |
| 189 | pericard*.ab,ti,kw. | 78,551 |
| 190 | isch?em*.ab,ti,kw. | 702,018 |
| 191 | emboli*.ab,ti,kw. | 242,201 |
| 192 | arrhythmi*.ab,ti,kw. | 180,615 |
| 193 | thrombo*.ab,ti,kw. | 692,395 |
| 194 | atrial fibrillat*.ab,ti,kw. | 173,332 |
| 195 | tachycardi*.ab,ti,kw. | 120,418 |
| 196 | endocardi*.ab,ti,kw. | 84,480 |
| 197 | (sick adj sinus).ab,ti,kw. | 3,876 |
| 198 | Stroke'/ | 212,103 |
| 199 | (stroke or stokes).ab,ti,kw,tw. | 532,949 |
| 200 | cerebrovasc*.ab,ti,kw. | 111,371 |
| 201 | cerebral vascular.ab,ti,kw. | 10,883 |
| 202 | apoplexy.ab,ti,kw. | 4,936 |
| 203 | (brain adj2 accident*).ab,ti,kw. | 297 |
| 204 | ((brain* or cerebral or lacunar) adj2 infarct*).ab,ti,kw. | 48,182 |
| 205 | Hyperlipidemias'/ | 50,247 |
| 206 | hyperlipid*.ab,ti,kw. | 65,392 |
| 207 | hyperlip?emia*.ab,ti,kw. | 4,419 |
| 208 | hypercholesterol*.ab,ti,kw. | 60,706 |
| 209 | hypercholester?emia*.ab,ti,kw. | 1,345 |
| 210 | hyperlipoprotein?emia*.ab,ti,kw. | 6,262 |
| 211 | hypertriglycerid?emia*.ab,ti,kw. | 23,832 |
| 212 | Arteriosclerosis'/ | 41,359 |
| 213 | Cholesterol'/ | 261,452 |
| 214 | cholesterol.ab,ti,kw. | 397,027 |
| 215 | or/1-78 | 4,553,662 |
| 216 | or/181-214 | 5,364,254 |
| 217 | 180 and 215 and 216 | 5,464 |

MEDLINE SEARCH

| **Query** | **Results from 8 Jan 2024** |  |
| --- | --- | --- |
| 1 | Patient Care Management/ | 4,747 |
| 2 | Comprehensive Health Care/ | 6,807 |
| 3 | Nursing Process/ | 7,433 |
| 4 | exp Nursing Assessment/ | 33,070 |
| 5 | Patient Care Planning/ | 39,558 |
| 6 | Case Management/ | 10,684 |
| 7 | delivery of Health care/ | 120,366 |
| 8 | Delivery of Health Care, Integrated/ | 14,388 |
| 9 | exp Managed Care Programs/ | 40,338 |
| 10 | Disease Management/ | 43,062 |
| 11 | exp Patient Care Team/ | 73,083 |
| 12 | exp Primary Health Care/ | 194,547 |
| 13 | Reminder Systems/ | 3,811 |
| 14 | Guideline Adherence/ | 35,178 |
| 15 | Home Care Services/ | 36,621 |
| 16 | Home Nursing/ | 8,668 |
| 17 | exp Nursing Services/ | 52,474 |
| 18 | exp Professional Role/ | 89,508 |
| 19 | Community Health Services/ | 33,234 |
| 20 | Medical Records/ or Medical Records Systems, Computerized/ | 85,043 |
| 21 | exp Patient Compliance/ | 86,188 |
| 22 | Life Style/ | 64,427 |
| 23 | Health Promotion/ | 82,135 |
| 24 | Health Services Administration/ | 4,555 |
| 25 | Education, Medical, Continuing/ | 25,624 |
| 26 | Marketing of Health Services/ | 14,841 |
| 27 | Patient Participation/ | 29,701 |
| 28 | Quality of Health Care/ | 77,811 |
| 29 | Quality Assurance, Health care/ | 56,932 |
| 30 | Exercise/ or Physical Fitness/ | 166,832 |
| 31 | Smoking Cessation/ | 33,117 |
| 32 | Diet/ or Fat-restricted/ or Carbohydrate-Restricted/ | 189,578 |
| 33 | Alcohol Drinking/pc [Prevention & Control] | 4,995 |
| 34 | Health Education/ | 64,004 |
| 35 | Community Health Planning/ | 5,247 |
| 36 | Communication/ or Communication Barriers/ or Information Dissemination/ | 127,364 |
| 37 | Nurse Clinicians/ | 8,561 |
| 38 | Nurse Practitioners/ | 19,012 |
| 39 | Risk Reduction Behavior/ | 14,322 |
| 40 | Pamphlets/ | 4,141 |
| 41 | Health Behavior/ | 57,043 |
| 42 | Health Knowledge, Attitudes, Practice/ | 127,618 |
| 43 | Secondary Prevention/ | 22,783 |
| 44 | Preventive Health Services/ | 14,501 |
| 45 | manag$adj3 care.tw. | 0 |
| 46 | (management adj3 program$).tw. | 23,711 |
| 47 | (case adj3 manag$).tw. | 25,177 |
| 48 | (patient adj3 management).tw. | 48,196 |
| 49 | (home adj3 intervention$).tw. | 4,475 |
| 50 | (home adj visit$).tw. | 11,069 |
| 51 | (discharg$ adj3 program$).tw. | 1,384 |
| 52 | (practice adj guideline$).tw. | 33,801 |
| 53 | (discharg$ adj3 plan$).tw. | 6,110 |
| 54 | (comprehensive adj3 care).tw. | 13,718 |
| 55 | (treatment adj3 plan$).tw. | 97,396 |
| 56 | (nurse$ adj3 led).tw. | 6,105 |
| 57 | (disease adj management).tw. | 21,859 |
| 58 | multidisciplin$.tw. | 122,824 |
| 59 | secondary prevention clinic$.tw. | 69 |
| 60 | reminder$.tw. | 16,818 |
| 61 | recall$.tw. | 90,685 |
| 62 | (nurse adj3 clinic$).tw. | 7,801 |
| 63 | (secondary prevention adj3 intervention$).tw. | 532 |
| 64 | (secondary prevention adj3 program$).tw. | 782 |
| 65 | Appointments.mp. and Schedules/ [mp=title, book title, abstract, original title, name of substance word, subject heading word, floating sub-heading word, keyword heading word, organism supplementary concept word, protocol supplementary concept word, rare disease supplementary concept word, unique identifier, synonyms, population supplementary concept word, anatomy supplementary concept word] | 9,888 |
| 66 | appointment$.tw. | 34,030 |
| 67 | (outreach adj nurs$).tw. | 104 |
| 68 | (outreach adj visit$).tw. | 339 |
| 69 | (lifestyle adj3 intervention$).tw. | 12,497 |
| 70 | (nurs$ adj intervention$).tw. | 8,648 |
| 71 | (education$ adj program$).tw. | 51,849 |
| 72 | (physical adj (activit$ or exercise$)).tw. | 171,361 |
| 73 | (exercise adj3 (train$ or intervention$ or program$ or activit$ or regim$)).tw. | 62,989 |
| 74 | fitness.tw. | 97,531 |
| 75 | (risk factor$ adj3 (modif$ or reduc$ or manage$ or monitor$ or self manage$)).tw. | 33,546 |
| 76 | (guideline directed medical therap* or GDMT).tw. | 1,081 |
| 77 | (guideline directed medical therap* or GDMT).mp. | 1,210 |
| 78 | (medic* adj3 uptit*).mp. | 33 |
| 79 | exp "Africa South of the Sahara"/ | 264,476 |
| 80 | ("Africa South of the Sahara" or sub-Saharan Africa or subSaharan Africa).ti,ab. | 30,139 |
| 81 | Central Africa.tw. | 3,865 |
| 82 | Eastern Africa.tw. | 1,317 |
| 83 | Southern Africa.tw. | 5,428 |
| 84 | Western Africa.tw. | 1,070 |
| 85 | Angola/ | 1,158 |
| 86 | Angola.ti,ab. | 1,779 |
| 87 | Cameroon/ | 6,649 |
| 88 | (Cameroon or Kamerun or Cameroun).ti,ab. | 8,838 |
| 89 | Cape Verde/ | 259 |
| 90 | (Cape Verde or Cabo Verde).ti,ab. | 790 |
| 91 | Comoros/ | 384 |
| 92 | (Comoros or Glorioso Islands or Mayotte).ti,ab. | 745 |
| 93 | Congo/ | 2,031 |
| 94 | (Congo not ((Democratic Republic adj3 Congo) or congo red or crimean-congo)).ti,ab. | 3,043 |
| 95 | Cote d'Ivoire/ | 3,614 |
| 96 | (Cote d'Ivoire or Cote dIvoire or Ivory Coast).ti,ab. | 4,594 |
| 97 | Eswatini/ | 783 |
| 98 | (eSwatini or Swaziland).ti,ab. | 1,207 |
| 99 | Ghana/ | 11,542 |
| 100 | (Ghana or Gold Coast).ti,ab. | 15,785 |
| 101 | Kenya/ | 19,930 |
| 102 | (Kenya or East Africa Protectorate).ti,ab. | 23,307 |
| 103 | Lesotho/ | 542 |
| 104 | (Lesotho or Basutoland).ti,ab. | 985 |
| 105 | Mauritania/ | 509 |
| 106 | Mauritania.ti,ab. | 764 |
| 107 | Nigeria/ | 34,729 |
| 108 | Nigeria.ti,ab. | 37,272 |
| 109 | (Sao Tome adj2 Principe).ti,ab. | 204 |
| 110 | Senegal/ | 6,286 |
| 111 | Senegal.ti,ab. | 6,685 |
| 112 | Sudan/ | 5,231 |
| 113 | (Sudan not South Sudan).ti,ab. | 8,920 |
| 114 | Zambia/ | 5,631 |
| 115 | (Zambia or Northern Rhodesia).ti,ab. | 6,908 |
| 116 | Zimbabwe/ | 6,651 |
| 117 | (Zimbabwe or Southern Rhodesia).ti,ab. | 7,059 |
| 118 | Botswana/ | 2,234 |
| 119 | (Botswana or Bechuanaland or Kalahari).ti,ab. | 3,316 |
| 120 | Equatorial Guinea/ | 322 |
| 121 | (Equatorial Guinea or Spanish Guinea).ti,ab. | 523 |
| 122 | Gabon/ | 1,647 |
| 123 | (Gabon or Gabonese Republic).ti,ab. | 2,065 |
| 124 | Mauritius/ | 631 |
| 125 | (Mauritius or Agalega Islands).ti,ab. | 1,192 |
| 126 | Namibia/ | 1,331 |
| 127 | (Namibia or German South West Africa).ti,ab. | 2,086 |
| 128 | South Africa/ | 50,219 |
| 129 | (South Africa or Cape Colony or British Bechuanaland or Boer Republics or Zululand or Transvaal or Natalia Republic or Orange Free State).ti,ab. | 45,395 |
| 130 | Benin/ | 1,963 |
| 131 | (Benin or Dahomey).ti,ab. | 4,288 |
| 132 | Burkina Faso/ | 4,054 |
| 133 | (Burkina Faso or Burkina Fasso or Upper Volta).ti,ab. | 5,451 |
| 134 | Burundi/ | 731 |
| 135 | (Burundi or Ruanda-Urundi).ti,ab. | 1,156 |
| 136 | Central African Republic/ | 850 |
| 137 | (Central African Republic or Ubangi-Shari).ti,ab. | 1,198 |
| 138 | Chad/ | 832 |
| 139 | Chad.ti,ab. | 1,538 |
| 140 | Democratic Republic of the Congo/ | 5,134 |
| 141 | (((Democratic Republic or DR) adj2 Congo) or Congo-Kinshasa or Belgian Congo or Zaire or Congo Free State).ti,ab. | 5,566 |
| 142 | Eritrea/ | 427 |
| 143 | Eritrea.ti,ab. | 725 |
| 144 | Ethiopia/ | 20,052 |
| 145 | (Ethiopia or Abyssinia).ti,ab. | 28,709 |
| 146 | Gambia/ | 2,736 |
| 147 | Gambia.ti,ab. | 2,788 |
| 148 | Guinea/ | 1,339 |
| 149 | (Guinea not (New Guinea or Guinea Pig* or Guinea Fowl or Guinea-Bissau or Portuguese Guinea or Equatorial Guinea)).ti,ab. | 3,350 |
| 150 | Guinea-Bissau/ | 1,040 |
| 151 | (Guinea-Bissau or Portuguese Guinea).ti,ab. | 1,195 |
| 152 | Liberia/ | 1,415 |
| 153 | Liberia.ti,ab. | 1,952 |
| 154 | Madagascar/ | 3,951 |
| 155 | (Madagascar or Malagasy Republic).ti,ab. | 5,774 |
| 156 | Malawi/ | 6,853 |
| 157 | (Malawi or Nyasaland).ti,ab. | 9,227 |
| 158 | Mali/ | 2,742 |
| 159 | Mali.ti,ab. | 4,456 |
| 160 | Mozambique/ | 3,101 |
| 161 | (Mozambique or Mocambique or Portuguese East Africa).ti,ab. | 4,748 |
| 162 | Niger/ | 1,443 |
| 163 | (Niger not (Aspergillus or Peptococcus or Schizothorax or Cruciferae or Gobius or Lasius or Agelastes or Melanosuchus or radish or Parastromateus or Orius or Apergillus or Parastromateus or Stomoxys)).ti,ab. | 4,190 |
| 164 | Rwanda/ | 3,220 |
| 165 | (Rwanda or Ruanda).ti,ab. | 4,302 |
| 166 | Sierra Leone/ | 1,982 |
| 167 | (Sierra Leone or Salone).ti,ab. | 2,972 |
| 168 | Somalia/ | 1,930 |
| 169 | (Somalia or Somaliland).ti,ab. | 2,054 |
| 170 | South Sudan/ | 285 |
| 171 | South Sudan.ti,ab. | 824 |
| 172 | Tanzania/ | 14,170 |
| 173 | (Tanzania or Tanganyika or Zanzibar).ti,ab. | 17,751 |
| 174 | Togo/ | 1,297 |
| 175 | (Togo or Togolese Republic or Togoland).ti,ab. | 1,847 |
| 176 | Uganda/ | 15,772 |
| 177 | Uganda.ti,ab. | 19,543 |
| 178 | Seychelles/ | 456 |
| 179 | Seychelles.ti,ab. | 905 |
| 180 | or/79-179 [ALL SUB-SAHARAN AFRICA COUNTRIES] | 362,146 |
| 181 | exp Cardiovascular Diseases/ | 2,756,349 |
| 182 | cardio*.tw. | 959,856 |
| 183 | cardia*.tw. | 726,042 |
| 184 | heart*.tw. | 983,247 |
| 185 | coronary*.tw. | 451,360 |
| 186 | angina*.tw. | 59,283 |
| 187 | ventric*.tw. | 466,771 |
| 188 | myocard*.tw. | 440,038 |
| 189 | pericard*.tw. | 49,107 |
| 190 | isch?em*.tw. | 451,130 |
| 191 | emboli*.tw. | 150,271 |
| 192 | arrhythmi*.tw. | 107,802 |
| 193 | thrombo*.tw. | 428,576 |
| 194 | atrial fibrillat*.tw. | 92,622 |
| 195 | tachycardi*.tw. | 69,202 |
| 196 | endocardi*.tw. | 56,518 |
| 197 | (sick adj sinus).tw. | 2,524 |
| 198 | exp Stroke/ | 177,624 |
| 199 | (stroke or stokes).tw. | 329,127 |
| 200 | cerebrovasc*.tw. | 67,450 |
| 201 | cerebral vascular.tw. | 6,613 |
| 202 | apoplexy.tw. | 3,536 |
| 203 | (brain adj2 accident*).tw. | 186 |
| 204 | ((brain* or cerebral or lacunar) adj2 infarct*).tw. | 32,485 |
| 205 | exp Hyperlipidemias/ | 71,182 |
| 206 | hyperlipid*.tw. | 37,442 |
| 207 | hyperlip?emia*.tw. | 2,546 |
| 208 | hypercholesterol*.tw. | 39,360 |
| 209 | hypercholester?emia*.tw. | 782 |
| 210 | hyperlipoprotein?emia*.tw. | 4,375 |
| 211 | hypertriglycerid?emia*.tw. | 15,237 |
| 212 | exp Arteriosclerosis/ | 201,349 |
| 213 | exp Cholesterol/ | 175,606 |
| 214 | cholesterol.tw. | 278,652 |
| 215 | or/181-214 | 4,543,941 |
| 216 | or/1-78 | 2,421,993 |
| 217 | 180 and 215 and 216 | 3,655 |
