# Supplementary material for: Implementation strategies to improve outcomes in patients with established cardiovascular disease in sub-Saharan Africa: A systematic review
Source: PLOS Glob Public Health. 2025 May 5;5(5):e0004544. doi: 10.1371/journal.pgph.0004544 (PMC12052185; doi:10.1371/journal.pgph.0004544)
Supplement: Attachment — Submitted filename: PGPH-D-24-01693-1 PLOS 2024 reviewed.docx [file pgph.0004544.s004.docx]

PLOS Global Public Health

# Implementation strategies to improve outcomes in patients with established cardiovascular disease in sub-Saharan Africa: a systematic review

--Manuscript Draft--

| Manuscript Number: | PGPH-D-24-01693 |
| --- | --- |
| Article Type: | Research Article |
| Full Title: | Implementation strategies to improve outcomes in patients with established cardiovascular disease in sub-Saharan Africa: a systematic review |
| Short Title: | Implementation strategies to improve outcomes in patients with cardiovascular disease |
| Corresponding Author: | Anoop S V Shah, MBChB  London School of Hygiene & Tropical Medicine Faculty of Epidemiology and Population Health  London, UNITED KINGDOM OF GREAT BRITAIN AND NORTHERN IRELAND |
| Order of Authors: | Leah Sanga |
|  | Anoop S V Shah |
|  | Jonathan A Hudson |
|  | Adrianna Murphy |
|  | Anthony Etyang |
|  | Alexander Perkins |
|  | Pablo Perel |
| Keywords: | Non-communicable diseases; Cardiovascular diseases; sub-Saharan Africa |
| Abstract: | Sub-Saharan Africa (SSA) is experiencing an epidemic of cardiovascular disease (CVD). Despite numerous evidence-based therapies and management guidelines for patients with acute or established CVD, significant gaps persist in their implementation in SSA. This systematic review aims to describe, synthesise and identify key gaps in the implementation strategies of evidence-based approaches that can improve clinical outcomes for patients with acute or established CVD in SSA.  We searched four databases for studies that examined the implementation strategies of evidence-based interventions for patients with acute or established CVD in SSA. Studies that did not focus on interventions were excluded. The primary outcome was major adverse cardiovascular events including myocardial infarction, stroke, cardiovascular death or hospitalisation. Secondary outcomes included adherence to treatment, improvement in modifiable risk factors, symptom measures, treatment complications, and psychosocial metrics, particularly those related to quality of life.  Nineteen studies met the inclusion criteria (nine studies evaluated patients with heart failure, 3 studies evaluated heart failure or ischaemic heart disease, 3 studies evaluated ischaemic heart disease and 4 studies evaluated stroke). Of the 19 studies, 5 evaluated task-sharing, 12 care models and 2 mobile health. The most common intervention strategies tested were in the field of cardiac rehabilitation. Only 3 studies (2 evaluating stroke and one heart failure) implemented an intervention in the in-patient setting with the rest evaluating startegies at discharge or in the ambulatory population. No studies evaluated implementation strategies in hospitalised patients with ischaemic heart disease.  This study highlights significant gaps in the implementation of cardiovascular disease interventions in SSA, particularly in the acute care settings and specifically in the field of implementation strategies for diagnostics and pharmacotherapeutic optimization. |
| Additional Information: |  |
| Question | Response |
| Financial Disclosure | This work was supported by the NIHR grant number GHR NIHR134544 |

| Enter a financial disclosure statement that describes the sources of funding for the work included in this submission and the role the funder(s) played. This includes grants and any commercial funding of the work or authors.  This statement will be typeset if the manuscript is accepted for publication.  *Please review the* [*submission guidelines*](http://journals.plos.org/globalpublichealth/s/submission-guidelines#loc-financial-disclosure-statement) *and the instructions link below for detailed requirements and guidance.* |  |
| --- | --- |
| Competing Interests  On behalf of all authors, disclose any competing interests that could be perceived to bias this work.  This statement will be typeset if the manuscript is accepted for publication.  *Please review the instructions link below and PLOS Global Public Health’s* [*competing interests*](https://journals.plos.org/globalpublichealth/s/competing-interests) *policy to determine what information must be disclosed at submission.* | None declared |
| Data Availability  Before publication, Authors are required to make fully available and without restriction all data underlying their findings. Please see our [PLOS Data](https://journals.plos.org/globalpublichealth/s/data-availability) [Policy](https://journals.plos.org/globalpublichealth/s/data-availability) page for detailed information on this policy.  A Data Availability Statement, detailing where the data can be accessed, is required at first submission. Insert your Data Availability Statement in the box below. | NA - literature review |

| Please see the [data reporting](https://journals.plos.org/globalpublichealth/s/submission-guidelines#loc-data-reporting) section of our submission guidelines for instructions on what you need to include in your Data Availability Statement.  This statement will be typeset if the manuscript is accepted for publication.  *PLOS allows rare exemptions to address legal and ethical concerns. If you have legal or ethical restrictions, please use the box below to detail these in full sentences for the Journal team to consider.* |  |
| --- | --- |

<https://www2.cloud.editorialmanager.com/pgph/download.aspx?id=384555&guid=ed37a435-d9ba-452c-bd4b-5bf499440589&scheme=1>Manuscript	[Click here to access/download;Manuscript;CVD SSA manuscript_final.pdf](https://www2.cloud.editorialmanager.com/pgph/download.aspx?id=384555&guid=ed37a435-d9ba-452c-bd4b-5bf499440589&scheme=1)

## Implementation strategies to improve outcomes in patients with established cardiovascular disease in sub-Saharan Africa: a systematic review

***Leah A Sanga^1^, Jonathan A Hudson^2^; Adrianna Murphy^3^, Anthony Etyang^4^; Alexander Perkins^1^; Pablo Perel; Anoop S V Shah^1,5^***

## Correspondence and requests for reprints:

Dr Anoop Shah,

Department of Non-communicable Disease Epidemiology, London School of Hygiene and Tropical Medicine,

Keppel Street, London, UK WC1E 7HT

Mobile: +(44) 7766 544 156

E-mail: [Anoop.Shah@lshtm.ac.uk](mailto:Anoop.Shah@lshtm.ac.uk)

**Affiliations**

^1^Department of Non-communicable Disease Epidemiology, London School of Hygiene & Tropical Medicine

^2^Kings College London BHF Centre, School of Cardiovascular and Metabolic Medicine & Sciences, London, UK

^3^Department of Health Services Research and Policy; London School of Hygiene & Tropical Medicine

^4^Dept of Epidemiology and Demography, KEMRI-Wellcome Trust Research Programme, Kilifi, Kenya

^5^Department of Cardiology, Imperial College NHS Trust, London, UK

## ABSTRACT

Sub-Saharan Africa (SSA) is experiencing an epidemic of cardiovascular disease (CVD). Despite numerous evidence-based therapies and management guidelines for patients with acute or established CVD, significant gaps persist in their implementation in SSA. This systematic review aims to describe, synthesise and identify key gaps in the implementation strategies of evidence-based approaches that can improve clinical outcomes for patients with acute or established CVD in SSA.

We searched four databases for studies that examined the implementation strategies of evidence-based interventions for patients with acute or established CVD in SSA. Studies that did not focus on interventions were excluded. The primary outcome was major adverse cardiovascular events including myocardial infarction, stroke, cardiovascular death or hospitalisation. Secondary outcomes included adherence to treatment, improvement in modifiable risk factors, symptom measures, treatment complications, and psychosocial metrics, particularly those related to quality of life.

Nineteen studies met the inclusion criteria (nine studies evaluated patients with heart failure, 3 studies evaluated heart failure or ischaemic heart disease, 3 studies evaluated ischaemic heart disease and 4 studies evaluated stroke). Of the 19 studies, 5 evaluated task- sharing, 12 care models and 2 mobile health. The most common intervention strategies tested were in the field of cardiac rehabilitation. Only 3 studies (2 evaluating stroke and one heart failure) implemented an intervention in the in-patient setting with the rest evaluating startegies at discharge or in the ambulatory population. No studies evaluated implementation strategies in hospitalised patients with ischaemic heart disease.

This study highlights significant gaps in the implementation of cardiovascular disease interventions in SSA, particularly in the acute care settings and specifically in the field of implementation strategies for diagnostics and pharmacotherapeutic optimization.

## INTRODUCTION

Sub-Saharan Africa (SSA) is experiencing an epidemic of cardiovascular disease (CVD).[1] Stroke, heart failure and ischaemic heart disease, the predominant pathologies constituting CVD, now make up an increasing proportion of acute admissions to hospitals in SSA.[2–4] Despite being nearly a decade younger, short and long-term outcomes following acute cardiovascular events are worse in SSA patients when compared to high-income countries.[5– 7] Individuals with acute or established CVD (stroke, heart failure or ischaemic heart disease) suffer from poor outcomes and significant economic impact making their effective management a public health priority.[8–10] The NCD Countdown 2030 Health Policy paper identifies the management of patients with acute or established cardiovascular disease as crucial interventions. These are prioritized to achieve the United Nations Sustainable Development Goal target 3.4, aiming to reduce premature mortality from non-communicable diseases by a third.[11]

Management of acute and established CVD consists of both pharmacological and non- pharmacological (for example dietary changes, physical activity, lifestyle management and cardiac rehabilitation) approaches to mitigate future cardiovascular morbidity and mortality.[12] These interventions aim to prevent the progression, avoid complications, and maximise the function and quality of life of individuals.[13] Despite extensive guidance, based on evidence-based approaches, for patients with acute or established CVD, significant gaps persist in their implementation in SSA. In particular, prescription of and adherence to proven treatments following cardiovascular events are suboptimal in SSA[5,6,14], and much lower than other regions of the world.[15,16]

Although research evaluating strategies to improve implementation of evidence-based interventions for primary prevention of cardiovascular disease in SSA has been increasing, similar research for managing patients with established CVD remains limited.[17,18]^,^[19] This systematic review aims to describe, synthesise and identify key gaps in the implementation strategies of evidence-based pharmacological and non-pharmacological approaches that improve clinical outcomes for patients with acute or established CVD in SSA.

## METHODS

***Data sources and search strategy***

The study was reported in accordance with the Preferred Reporting Items for Systematic Reviews and Meta-Analyses (PRISMA) guidelines.[20] We searched MEDLINE, EMBASE, Global Health, and Google Scholar for studies that examined the implementation strategies of evidence-based therapy for patients with acute or established CVD in SSA. We manually searched bibliographic references of key papers. Our search strategy utilised the keywords: “cardiovascular disease”, “prevention interventions”, and “sub-Saharan Africa”. We included studies of any design, in any language, published from database inception up to the 31^st^ of December, 2023. The full search criteria is provided in the supplement.

***Eligibility criteria***

*Inclusion criteria*

We included studies reporting on interventions for managing acute or established CVD among adults (>=18 years) in the SSA region. Cardiovascular disease was restricted to the following pathologies: stroke, heart failure or ischaemic heart disease. Clinical trials, non randomised clinical studies, pre and post-intervention studies, case-control studies, and cross-sectional studies were eligible for inclusion. We did not impose restrictions based on specific definitions or diagnostic criteria of cardiovascular diseases to ensure inclusion of studies that may not have explicitly detailed their criteria

*Exclusion criteria*

Studies that did not focus on interventions were excluded.

***Outcome***

***The primary outcome was major adverse cardiovascular events, which included recurrent myocardial infarction, stroke, cardiovascular death, and hospitalisation due to heart failure or other cardiovascular causes. Given the expected heterogeneity of studies and diverse outcomes, secondary outcomes were also assessed. These included adherence to treatment, improvement in modifiable risk factors, hospitalizations, symptom measures, treatment complications, and psychosocial metrics, particularly those related to quality of life.***

***Study selection***

Two reviewers (LS & JH) independently screened and assessed retrieved studies for eligibility based on the inclusion and exclusion criteria. Conflicts were resolved through consensus or consultation with a third reviewer (AS). Data, including study characteristics and baseline population details, were extracted by one investigator (LS) and verified by another (JH). All studies identified were imported to Rayyan[21], an internet-based program that facilitates collaboration between investigators during the screening and selection of studies to be finally included in the review. Duplicates were removed and studies screened on the Rayyan platform.

***Evidence synthesis and risk of bias***

A narrative synthesis was conducted to provide an overview of the findings from the included studies, focusing on the type and effectiveness of implementation strategies to improve outcomes in patients with CVD in SSA.

We evaluated the reporting and methodological quality of the included studies using the Evidence project risk of bias tool.[22] This tool includes eight items that award one point for the following items: (1) prospective cohort; (2) control or comparison group; (3) pre/post intervention data; (4) random assignment of participants to the intervention; (5) random selection of subjects for assessment; (6) follow-up rate of 80% or more; (7) comparison groups equivalent on socio-demographic measures; and (8) comparison groups equivalent

at baseline on outcome measures.

## RESULTS

***Review of search results***

The database search yielded 14,429 results and 6 additional studies were identified from bibliographic references giving a total of 14,435 studies reviewed. After screening 34 studies were read in detail to assess eligibility. Of these 19 met the inclusion criteria and were included in the final analysis (Fig 1).

***Figure 1:*** *PRISMA flow chart of studies identification, screening, and inclusion*

***Characteristics of the studies included***

*Geographical distribution:* Out of the 19 studies included, one study (5.3%) was international, encompassing three SSA countries[23] . The remaining studies were conducted in single countries as follows: five studies (26.3%) in South Africa[24–28], four (21%) in Nigeria[29–32], two (10.5%) in Ghana[33,34] and Ethiopia[35,36], one (5.3%) in Uganda[37], Sudan[38], Rwanda[39], Kenya[40] and Benin[41] (Table 1 & Fig 2)

***Figure 2:*** *Cartogram highlighting countries contributing studies to this systematic review. The colors illustrate the numerical scale for the number of studies. The color box in the legend summarises the number of studies with the number in the squared brackets summarising the number of countries.*

***Table 1****: Summary of Study Characteristics*

| **Author, Year, Country** | **Design and setting** | **Population** | **Intervention** | **Implementa tion strategy** | **Comparator** | **Sample size** | **Mean age** | **Female (%)** | **Outcome measure(s)** | **Measurement time points** | **Effects of the intervention** |
| --- | --- | --- | --- | --- | --- | --- | --- | --- | --- | --- | --- |
| **Heart Failure** |  |  |  |  |  |  |  |  |  |  |  |
| Anane et al, 2013[34];  Ghana | Pre-post intervention study; Teaching hospital | Adult patients ≥ 30 years discharged from acute ward with clinical criteria for HF | Pharmacist led counselling sessions involving education on medical risk management, health behaviour change, and medication compliance. | Task sharing | No | 583 | nr | nr | All cause and all cause re- hospitalization | 6 months | Low mortality rate (1.2%) and low rate of rehospitalization (2.4%) following the 6 month follow up period. |
| Eberly et al, 2019[39];  Rwanda | Retrospective cohort; Rural district level hospital | Adult HF patients  ≥18 years referred to the clinic on discharge from hospital with cardiologist | Nurses-led delivery care model for heart failure management with monthly supervision from cardiologist. | Task sharing | N/A | 719 | nr | 72 | All-cause mortality | 5 years | 5 year event rate in adults was 57.4%.  Improvement in 5-year mortality over time: 38.8% in the first 5 year period (2006-2011) and 27.1% in |

|  |  | confirmed diagnosis. |  |  |  |  |  |  |  |  | the second 5-year period (2012 to 2017) |
| --- | --- | --- | --- | --- | --- | --- | --- | --- | --- | --- | --- |
| Wondesen A et al, 2022[35];  Ethiopia | Pre-post intervention study; Tertiary care hospital | Ambulatory HF  patients ≥ 18 years | Pharmacist and nurse led, providing information and educational material on self-care, physical activity, and medication adherence. Pharmacists identified and resolved drug therapy problems. | Task sharing | No | 412 | 44.57 | 53.6 | - Drug therapy problem- categorised as adverse drug reactions, unnecessary drug therapy, need for additional drug therapy, dosage too low, dosage too high, ineffective drug and non-adherence. - Medication adherence - Treatment satisfaction | 6 months | Significantly reduced drug therapy problems, improved medication adherence, and increased treatment satisfaction. |
| Ahmed et al., 2021[38];  Sudan | Pre-post intervention study; Tertiary care hospital | Ambulatory HF patients ≥ 18 years with documented LVEF≤40% | Pharmacist led management of heart failure including initiation, up titration, and changing between drug classes. | Task sharing | No | 110 | 55.8 | 42.7 | Achievement of target doses of GDMT for heart failure. | 6 months | Significant in the proportion of patients achieving target doses of GDMT and improvement in LVEF. |

| Dessie et al., 2021[36];  Ethiopia | Cluster randomized control trial; Tertiary care hospital | Ambulatory patients with chronic heart failure diagnosed by imaging or NYHA class. | Nurse led heart failure self-care educational program comprising of intensive four-day training followed by one-day sessions offered every four months. Education references leaflets distributed to aid in- home care. | Task sharing | Yes, Patients from control cluster received usual care | 219 | nr | 47.7  interve ntion 68.4  control | Hear failure self-care adherence measured by Medical Outcomes Study- Specific Adherence Scale | Baseline, 4  months, 8  months, 12 months | Significantly higher self-care adherence scores observed in the intervention group after two and three rounds of educational sessions. |
| --- | --- | --- | --- | --- | --- | --- | --- | --- | --- | --- | --- |
| Mebazaa et al., 2022[23];  Mozambiqu e, Nigeria, South Africa | Randomised controlled trial; Setting not reported | Hospiltalised patients with acute heart failure and NT-proBNP  >2500 pg/mL. Not on optimal GDMT. | Risk startifictaion and post-discharge care based of optimisation of heart failure therapies based on an algorithm including NT-proBNP diagnostics | Model of care | Yes, Patients receiving usual care | 1078 | 63 | 39 | 180 day heart failure readmission or all cause death | 180 days | Significant reduction in heart failure readmission or all cause death (15.2% vs. 23.3%, RR 0.66 (0.5 to 0.86)  p= 0.0021) |

| Ajiboye et al, 2015[32];  Nigeria | Randomized controlled trial; Teaching hospital | Ambulatory HF patients with LVEF  ≤ 40%. | Supervised exercise training: a combination of aerobic and resistance exercises, performed three times a week for 12 weeks. | Model of care | Yes Control group received standard medical care | 51 | 54 | nr | 6 minute walk test | 12 weeks | Significant improvement in  6MWD (Δ+34.7m vs. Δ-  5.0m) and VO2 max  (Δ+8.1mL/kg/min vs. Δ- 0.8ml/Kg/min) in exercise group compared to control group. Significant improvements in resting heart rate, respiratory rate, and BMI in the exercise group. |
| --- | --- | --- | --- | --- | --- | --- | --- | --- | --- | --- | --- |
| Awotidebe et al., 2016[30];  Nigeria | Randomized controlled trial; Teaching hospital | Ambulatory adult HF patients ≥50 years, with NYHA class II and LVEF  ≤40% | Supervised cardiac rehabilitation exercises that included both aerobic and resistance exercises that increased in intensity weekly. | Model of care | Yes Participants randomly assigned to control group | 70 | 68.9  interve ntion 64.3  control | 48.6  interve ntion 60  control | (1) Hand grip strength, (2) 6 minute walk test to assess VO2 max, (3) activity of daily living questionnaire | 8 weeks | Significant improvements in ADL questionnaire, 6MWD (406.0m ± 29.7m vs. 321.0m  ± 25.7m), and VO2 max (10.3 ± 0.5mL/kg/min vs. 8.9 ± 0.4ml/kg/min) in the intervention group compared to the control group |

| Ajiboye et al., 2013[31];  Nigeria | Randomized controlled trial; Teaching hospital | Ambulatory HF patients with LVEF  ≤ 40% | Supervised cardiac rehabilitation: a combination of aerobic and resistance exercises, performed three times a week for 12 weeks. | Model of care | Yes, patients were monitored and received education and counselling sessions but not exercise training | 38 | 54.2 | 46.9 | Various cardiorespiratory factors: resting heart rate, blood pressure, respiratory rate, and SPO2 | 12 weeks | Exercise group showed significant reduction in resting heart rate, respiratory rate, and oxygen saturations. No improvement seen in the control group. Significant improvements seen in all body composition parameters (except BMI  )which met significance. |
| --- | --- | --- | --- | --- | --- | --- | --- | --- | --- | --- | --- |
| **Heart failure/ Ischaemic heart disease** | | | | | | | | | | | |

| Ngeno et al., 2022[40];  Kenya | Non randomised clinical study; Teaching hospital | Ambulatory HF patients ≥ 18 years with NYHA class II or III | Non randomly enrolled to institution-based supervised cardiac rehabilitation or home based cardiac rehabilitation.  Particpants in these two groups were compared to an observation group. | Model of care | Yes, enrolled to observation arm/control group | 100 | 51 | 28 | Protocol adherence along with self-reported functional capacity and 6 minute walk time distance | 1 month | Higher protocol adherence observed in the institution based rehabilitation group. All three arms showed significant improvement in NYHA class and 6 minute walk distance with no significant inter group differences. |
| --- | --- | --- | --- | --- | --- | --- | --- | --- | --- | --- | --- |
| Digenio A.G et al., 1996[28];  South Africa | Pre-post intervention study; Rehabilitation Centre | Discharged patients recovering from acute myocardial infarction and who had a left ventricular ejection fraction of 30% or less | Medically supervised exercise training programme (walking, jogging or cycling) | Model of care | No | 28 | 63.5+/-  7.5 | nr | Hemodynamic, left ventricular function and effort tolerance parameters | 6months | There was significant improvement in maximal exercise capacity and significant reduction in cardiovascular demands during submaximal exercise for patients who completed the training programme. |

| NiamaNata D, 2013[41];  Benin | Pre-post intervention study; Teaching hospital | Ambulatory patients diagnosed with heart failure, coronary artery disease, or hypertension. | Institution based supervised exercise training, 3 session a week for 10 weeks conducted predominantly on cycle ergometer or rowing machine. | Model of care | No | 27 | 45.8  +/- 11.1  CAD 43.25  +/- 8.8  HF  43 +/-  9.7  HTN | 26 | Various cardiorespiratory factors: resting heart rate, blood pressure, and BMI | 10 weeks | Significant reduction in HR  Δ-17.5bpm, SBP Δ-  14.7mmHg, DBP Δ-6mmHg, BMI Δ-2.2kg/m2. |
| --- | --- | --- | --- | --- | --- | --- | --- | --- | --- | --- | --- |
| Ischaemic heart disease | | | | | | | | | | | |
| van Rooy & Y Coopoo, 2017[24];  South Africa | Pre-post intervention study; Rehabilitation Centre | Ambulatory patients who underwent coronary artery bypass grafting aged between 40  and 80 years. | Supervised Individualized exercise programmes  Lifestyle manual | Model of care | No | 18 | nr | 5.6 | Cardiovascular disease risk Change in lifestyle habits, nutrition knowledge and physical activity profile | 12 weeks | Significant improvements were noted in the alterable risk score, total risk, body fat percentage, diet, regular exercise and stress levels The intervention significantly reduced all evaluated indices of |

|  |  |  |  |  |  |  |  |  |  |  | cardiovascular disease risk and increased physical activity levels |
| --- | --- | --- | --- | --- | --- | --- | --- | --- | --- | --- | --- |
| Morris et al., 1993[27];  South Africa | Pre-post intervention study; Rehabilitation Centre | Ambulatory patients with coronary artery disease- 41.6% post coronary artery bypass grafting  , 33.8% post myocardial infarction | Six month cardiac rehabilitation programme consisting of aerobic exercise sessions three times per week and weekly discussion about lifestyle modification. | Model of care | No | 108 | 57 +/-2 | 12 | apo AI increased by 2.73% (p- value 0.0198), Lp(a) increased by 2.23% (p-value 0.0353). No statistically significant change in apo B | 6 months | For every ml/kg/min increase in peak Vo2, it is estimated  that the percentage change in TC decreases by 0.95%. |
| Joughin et al., 1999[26];  South Africa | Non randomised clinical study; Rehabilitation Centre | Ambulatory patients with coronary heart disease with recent myocardial infarction, | Endurance training: combination of walking and jogging on a measured outdoor circuit or cycling on a stationary ergometer. | Model of care | Yes Assignment to control group based on attendance, | 111 | 57.3+/-  108  interve ntion | 8.6  interve ntion 5.5  control | Haemodynamic, electrocardiographic and metabolic measurements at rest, submaximal and at peak exercise level | 6month and 18month after intervention | Significant decrease in resting heart rate and BP, Submaximal heart rate, BP, rate-pressure product and ventilation and Rate- |

|  |  | coronary artery bypass grafting or percutaneous coronary intervention | Compliers allocated to intervention group were compared to non- compliers/drop-outs allocated to control group |  | patients attending less than 10%  sessions and dropped out for non- medical reasons formed control group |  | 59.4+/-  10.5  control |  |  |  | pressure product for compliers  Significant increase in Ventilatory threshold, Peak oxygen uptake, heart rate and ventilation and Treadmill time  No significant change on the control group except for increase in ST-segment depression on the exercise ECG |
| --- | --- | --- | --- | --- | --- | --- | --- | --- | --- | --- | --- |
| **Stroke** | | | | | | | | | | | |
| Nakibuuka et al., 2016[37];  Uganda | Non randomised clinical study; Tertiary care hospital | Hospitalised patients with confirmed stroke | 72-hour stroke care bundle including: rapid initial stroke screening, brain CT scan, bedside swallow evaluation, aspirin administration, physiological monitoring | Model of care | Yes Non-  randomized control patients received usual | 254 | nr | nr | Mortality within 30 days from stroke onset, median length of stay, in hospital mortality, median survival time, functional outcome at 30 days | 30 days post stroke | No difference in mortality nor functional outcomes. |

|  |  |  | and management, and early rehabilitation |  | standard care |  |  |  |  |  |  |
| --- | --- | --- | --- | --- | --- | --- | --- | --- | --- | --- | --- |
| Sarfo et al., 2019[33];  Ghana | Cluster randomized controlled trial; Teaching hospital | Ambulatory patients with recent stroke and uncontrolled hypertension (SBP  ≥ 140mmHg) | Mobile Health nurse guided intensive home blood pressure monitoring with the use of Bluetooth blood pressure device and smartphone. | Mobile health | Yes Randomized control selection, unmasked, control received text message on healthy lifestyle behaviour but not medication adherence | 60 | 54.3  interve ntion 55.9  control | 40  interve ntion 30  control | Blood pressure control defined as a blood pressure < 140/90 mmHg at 9 months | 9 months | Study showed feasibility and signal of improvement in blood pressure control among stroke survivors in a resource-limited setting via an mHealth intervention. The proportion of participants with systolic BP<140mmHg was significantly higher in the intervention group. |

| Owolabi et al., 2019[29] ;  Nigeria | Randomized controlled trial; Teaching and district hospitals | Ambultaory patients with recent stroke within the last year. | (1) Pre appointment text message reminders and post appointment text message summaries (2) Disease self- management support including risk factor control monitoring with custom report cards and educational videos (3) Electronic outpatient stroke registry | Mobile health | Yes Controls randomly assigned, received standardixe d version of usual care and provided with name and contact information of a phone contact | 400 | 57.2 | 36.5 | Mean change in systolic blood pressure | 12 months | No significant difference in SBP reduction between the two groups. |
| --- | --- | --- | --- | --- | --- | --- | --- | --- | --- | --- | --- |
| De Villiers et al., 2009[25];  South Africa | Pre-post intervention study; Hospital setting | Hospitalised patients with a clinical diagnosis of stroke | Stroke unit implementation delivering multidisciplinary stroke care including: | Model of care | No | 195 | 58.8 | 60 | (1) Length of hospital stay, (2)  in-hospital mortality, (3) transfer to referral hospital, (4) number of patients who had CT brain scans performed | 3 months pre- intervention  3 months post- intervention | Decrease in in-hospital mortality (33% to 16%) Significant increase in hospital stay (mean of 5.1to  6.8 days |

|  |  |  | (1) Stroke treatment guidelines (2) Twice weekly stroke ward rounds including physiotherapists and social workers |  |  |  |  |  |  |  | Significant increase in referral to inpatient rehabilitation (5% to 19%) No significant increase in number of CT brain scans and number of referrals to tertiary facility |
| --- | --- | --- | --- | --- | --- | --- | --- | --- | --- | --- | --- |

***Study setting and population***

All studies recruited participants from either hospital outpatient or inpatient medical facilities with none conducted in community settings. Four studies (21.1%) included participants with stroke[25,29,33,37]; nine (47.4%) with heart failure[23,30–32,34–36,38]; three (15.8%) with ischaemic heart disease[24,26,27]; and three (15.8%) included patients with either ischaemic heart disease or heart failure[28,40,41] (Table 1). A total of 4397 participants were included, with individual study sizes ranging from 18 to 1078 participants. The mean age of participants ranged from 43 to 69 years of age, and the proportion of women included ranged from 5.5% to 68.4%. Out of 19 studies, five (62.5%) were indivual participant level randomised controlled trials[^18,24–27^](https://www.zotero.org/google-docs/?nTYhet), two were cluster randomised trials[33,36], three were non-randomised clinical studies[26,37,40], eight were pre-post intervention studies and one was a retrospective study.[24,25,27,28,34,35,38,41]

Only three studies (one evaluating heart failure[23] and two studies evaluating stroke[25,37]) recruited participants in the acute setting with the remaining involving patients post discharge / community ambulatory patients. Eleven studies (57.9%) were conducted in either tertiary or teaching hospitals, one study (5.3%) was conducted across tertiary and district-level hospitals[29] and four studies were conducted in rehabilitation centres. [24,26–28] Only one study (5.3%) was specifically conducted in a rural setting[39], and two studies (10.5%) did not report the healthcare facility level.[23,25]

***Study quality***

The assessment of the quality of studies is summarised in Table 2. Of the 19 studies which were included, five were individual participant level randomised controlled trials[23,29–32];

two were cluster randomised trials[33,36]; three were non-randomised clinical studies[26,37,40]; eight were pre-post intervention studies[24,25,27,28,34,35,38,41]; and one was a retrospective study.[39]

**Table 2**: Quality Assessment of included studies

| **Reference and country** | **Design** | **coho rt** | **control or compari son group** | **pre/post intervent ion data** | **random assignme nt of participa nts to the intervent ion** | **random selection of participa nts for assessm ent** | **follo w up rate of**  **>80**  **%** | **comparison groups equivalent on sociodemogra phic** | **compari son groups equivale nt at baseline on outcome measure**  **s** | **Sco re** |
| --- | --- | --- | --- | --- | --- | --- | --- | --- | --- | --- |
| Nakibuuka et al. 2016 Uganda | Non randomise d clinical  study | yes | yes | na | no | yes | yes | yes | na | 5 |
| Ahmed et al. 2021  Sudan | Pre-post  interventi on study | yes | no | yes | no | no | nr | na | na | 2 |
| Wondese n A et al 2022  Ethiopia | Pre-post interventi on study | yes | no | yes | yes | yes | yes | yes | na | 6 |
| Dessie et al 2021 Ethiopia | Cluster randomise d control  trial | yes | yes | yes | yes | no | yes | no | yes | 6 |
| Sarfo et al 2015  Ghana | Cluster randomise d control  trial | yes | yes | yes | yes | no | yes | yes | yes | 7 |

| Anane et  al 2013 Ghana | Pre-post  interventi on study | yes | no | yes | no | no | yes | na | na | 3 |
| --- | --- | --- | --- | --- | --- | --- | --- | --- | --- | --- |
| Ngeno et al 2022 Kenya | Non randomise d clinical  study | yes | yes | no | no | no | yes | no | no | 3 |
| Eberly et al 2019 Rwanda | Retrospec tive review of interventi  on | yes | na | no | na | no | na | nr | nr | 1 |
| O.A  Ajiboye et  al 2015 Nigeria | Randomis ed controlled  trial | yes | yes | no | yes | no | no | yes | yes | 5 |
| Owolabi et al 2019 Nigeria | Randomis ed controlled  trial | yes | yes | yes | yes | no | yes | yes | yes | 7 |
| L. de Villiers et al 2009 South  Africa | Pre-post interventi on study | yes | no | yes | no | no | nr | no | nr | 2 |
| L Van Rooy & Y Coopoo 2017  South  Africa | Pre-post interventi on study | yes | no | yes | no | no | nr | na | na | 2 |
| T. O.  Awotideb e et al. | Randomis ed controlled  trial | yes | yes | no | yes | no | yes | no | yes | 5 |

| 2016  Nigeria |  |  |  |  |  |  |  |  |  |  |
| --- | --- | --- | --- | --- | --- | --- | --- | --- | --- | --- |
| Ajiboye et al 2013 Nigeria | Randomis ed controlled  trial | yes | yes | no | yes | no | yes | yes | yes | 6 |
| Kpadonou et al 2013  Benin | Pre-post interventi  on study | yes | no | yes | no | no | yes | na | na | 2 |
| Joughin et al 1999 South  Africa | Non randomise d clinical  study | yes | yes | yes | no | no | yes | no | no | 4 |
| Digenio et al 1996 South  Africa | Pre-post interventi on study | yes | no | yes | no | no | nr | na | na | 2 |
| Morris et al 1993 South  Africa | Pre-post interventi on study | yes | no | yes | no | no | nr | na | na | 2 |
| Mebazaa et al 2022 Mozambiq ue, Nigeria, South  Africa | Randomis ed controlled trial | yes | yes | yes | yes | no | yes | yes | yes | 7 |

All but four studies scored 3-5 out of 8 in the quality assessment. One study[39] scored 1 out of 8 and three studies[23,29,33] scored 7 out of 8. The three studies which scored 7 were all randomized controlled trials. Two of these studies[23,33] did not mask both the

participants and investigators to treatment allocation. Five studies[24,25,27,28,38] did not report on attrition and one study[32] had follow-up rate of less than 80%.

***Implementation strategies***

Across all three cardiovascular pathologies, different forms of implementation strategies were assessed. Five studies, all evaluating heart failure care, tested the role of task sharing with nurse- or pharmacy-led care delivery. Two studies, both evaluating stroke, tested the role of mobile health. Twelve studies evaluated care models.[23–28,30–32,37,40,41] Of these 9 were specific to cardiac rehabilitation in the context of heart failure or ischaemic heart disease.[24,26–28,30–32,40,41] The remaining three studies evaluated patients in the acute setting with two evaluating care models in stroke[25,37] and one in heart failure[23] and one evaluating multidisciplinary medical models in stroke.[29] These studies are discussed in detail, by cardiovascular pathology, below.

***Heart failure***

Twelve studies recruited patients with heart failure. Among these, nine studies (75%) specifically recruited patients with heart failure[23,30–32,34–36,38,39] and three studies included patients with heart failure or ischaemic heart disease.[28,40,41] Only one study^21^ targeted care delivery of acute heart failure, with the remaining designed to improve care for recently discharged or ambulatory patients with heart failure.

Intervention strategies varied, with ten (83%) studies implementing patient-targeted interventions and two targeting the healthcare system.[23,39] Of the patient-targeted interventions, three addressed medication compliance and up-titration in chronic heart

failure pharmacotherapy.[34,35,38] These included pharmacy and nurse-led interventions to target appropriate up-titration and ensure compliance of guideline directed medical therapy in ambulatory patients.[35,38] These studies showed either improved adherence and treatment satisfaction alongside an increase in the proportion of patients receiving goal directed medical therapy. One of these studies provided medical counselling to hospitalised patients with heart failure in Ghana, showing better clinical outcomes at 6 month follow up.[34]

Implementation of supervised cardiac rehabilitation care models was the focus of six of ten studies[27,30–32,40,41] with five implementing institution-based exercise programmes and one study from Kenya[40] comparing home-based with institution-based cardiac rehabilitation. All six studies reported improvements across a variety of physiological measures. Educational interventions were also prominent, with three of the patient-level interventions providing education about heart failure and support health-promoting behaviours.[23,35,36]

STRONG-HF, a large multinational randomised controlled recruited heart failure patients in South Africa, Mozambique, and Nigeria, implemented a care model which incorporated an intensive follow-up regimen post discharge for patients who had been admitted with acute heart failure.[23] The regimen was based on risk-stratification using circulating biomarkers of cardiac remodelling, and showed a significant improvement in a composite of all-cause mortality and heart failure rehospitalization. Another notable study from Rwanda targeted both healthcare providers and the healthcare system by training nurses in rural clinics to perform cardiac ultrasound and provide heart failure care. They demonstrated a reduction in mortality of their population over two five-year periods.[39]

***Stroke***

Studies recruiting patients with stroke evaluated implementation startegies in both the inpatient[25,37] and outpatient hospital settings.[29,33] In the inpatient setting, two studies implemented a form of structured stroke care. De Villiers *et al.* established a multi- disciplinary stroke unit within the medical ward engaging allied healthcare professionals and creating protocols to enhance stroke care.[25] This approach reduced inpatient mortality and increased referrals to rehabilitation centres. Conversely, Nakibuuka et al. implemented a stroke care bundle at a tertiary hospital in Uganda for acute stroke patients.[37] Despite implementing a comprehensive care model of diagnostics, pharmacological management and post-discharge care, there was no difference on inpatient stroke mortality, however, this may have been a result of greater stroke severity in patients enrolled during the intervention phase.

In the outpatient setting, two studies attempted to improve blood pressure control in stroke survivors.[29,33] The PINGS trials[33] implemented nurse-guided intensive home blood pressure monitoring facilitated by a smartphone application, while the THRIVES trial[29] employed pre-appointment SMS reminders, tailored disease self-management report cards, and educational videos. Both these interventions showed feasibility. The THRIVES trial did not demonstrate a significant improvement in blood pressure control, whilst the PINGS feasibility study did show a signal towards improvement in blood pressure control.

***Ischaemic heart disease***

Three studies[24,26,27] solely evaluated patients with ischaemic heart disease. All studies looked at cardiac rehabilitation care models and originated from South Africa, showing an improvement in haemodynamics and parameters of exertional tolerance. Our systematic

review did not find any studies evaluating care models for patients with ischaemic heart disease in the acute setting including those presenting with acute coronary syndrome.

## DISCUSSION

Our systematic review summarises the body of literature evaluating implementation strategies of evidence-based interventions for management of patients with CVD in SSA. We identified 19, of which five were individual participant level randomised controlled trials[23,29–32]; two were cluster randomised trials[33,36]; three were non-randomised clinical studies[26,37,40]; eight were pre-post intervention studies[24,25,27,28,34,35,38,41]; and one was a retrospective study.[39] Of the seven randomised trials, four evaluated cardiac rehabilitation care models in the context of heart failure, showing improvements in one or both of exertional tolerance and cardiac haemodynamics.[30–32,36] Mebazaa et al[23] showed a significant reduction in hospitalisation and mortality, following randomisation to biomarker-guided post discharge care. The remaining two randomised trials[29,33] were in patients with stroke using text messages to improve blood pressure control. Both showed feasibility with one study showing a signal towards better blood pressure control. Of the eight pre-post intervention studies, 4 were based on cardiac rehabilitation in patients with heart failure or coronary disease.[24,27,28,41] Similar to randomised trials[30,31,31,36], the majority of these showed improvement in extertional tolerance or haemodynamics. Two pre-post intervention studies were pharmacist led, showing improved medical adherence and increased proportion achieving target drug doses.[35,38]

Several important observations emerge from our review. Firstly, the majority of studies did not focus on patients in the acute or peri-discharge period. This is important, as the highest risk of morbidity and mortality is experienced by patients during this period, either as inpatients or shortly after discharge.[42] Secondly, studies specifically targeting

implementation strategies of pharmacotherapy, diagnostic tools, multidisplinary teams or risk stratification approaches in the acute setting were scarce; we found only one study for acute heart failure and two for stroke. We found none evaluating patients with acute coronary syndrome. Thirdly, only three studies[25,29,39] recruited patients from non- teaching or non-tertiary care settings. This finding has particular relevance to the region, given that nearly 6 out 10 people in SSA do not reside in urban regions.[43] The applicability of study findings from urban settings to rural ones will further be hindered given differences in health infrastructure and healthcare provider expertise. Finally, it was encouraging to observe that many studies did evaluate the implementation of cardiovascular rehabilitation programmes, an area that is generally given less importance.

Despite SSA bearing a significant and increasing global burden of CVD, its populations remain underrepresented in both evidence generation and implementation.[23,29–32] The prognosis following acute cardiac pathologies in SSA remains unacceptably poor, with a quarter of all adult hospitalizations in certain countries being cardiovascular-related. Despite being a decade younger on average compared to high-income countries, cardiac patients in SSA experience upto 3-fold higher short-term case fatality rates (30% vs. 9%).[9,44] Annually, low and middle-income countries witness an estimated 16 million excess deaths, with over a third attributed to cardiovascular pathology. Approximately 4.5 million of these deaths occur in the African region, predominantly due to inadequate secondary care health systems.[45] These deficiencies contribute as significantly to excess deaths as immature primary care systems in Africa. The high mortality, at least in part, may be exacerbated by poor links between different levels of care. Furthermore, despite the high burden of CVD and poor outcomes, research funding for the discipline has been disproportionately low.

Provision of appropriate pharmacotherapy across SSA patients with established cardiovascular disease remains poor. Only 1 in 10 patients with established CVD use aspirin, across many African countries[16], and less than 50% of heart failure patients receive appropriate management in Kenya[46]. Low uptake of appropriate pharmacotherapeutic and non-pharmacotherapeutic approaches across patients in SSA with established CVD is likely to be due to inadequacies across several implementation strategic domains.[47] These implementation strategies specifically tackle the ‘how to’ component for delivering optimal clinical care with recent guidance published on defining and operationalising these strategies.[17,18] These guidelines should be considered by implementation researchers and stakeholders aiming to improve care in patients with established CVD from SSA.

The lack of published literature, especially in the acute cardiovascular care setting, may reflect the current funding priorities in global cardiovascular health. For example, from2017- 2024, UK funding bodies, like the NIHR, allocated over £370 million in healthcare research, with less than 1% (~£2 million) directed towards management of patients with acute or established CVD compared to £40 million for primary cardiovascular care.[48] The mismatch between disease burden due to established cardiovascular disease and research funding underscores the importance of futher research in the area. In particular, efforts now need to concentrate on the optimal approaches in implementing evidence-based therapy for the three core cardiovascular pathologies. These efforts need to be contextualised to the settings, taking into account the differing healthcare infrastructure both within and

between countries across the African continent.

Several limitations of our work should be considered. The literature search was confined to four databases and excluded grey literature, potentially omitting relevant unpublished studies. Furthermore, our focus was restricted to specific cardiovascular pathologies, namely stroke, heart failure and ischaemic heart disease. Whilst these pathologies constitute the majority of all CVD, our review does not shed any light on gaps in the implementation strategies of other CVD that may be prevalent in the region..

## CONCLUSION

This evidence synthesis highlights significant gaps in the implementation strategies for manageing acute or established CVD in SSA. Particular gaps were highlighted in the acute care setting, specifically evaluating diagnsotics and pharmacotherapeutic optimizations. We also highlight a notable lack of studies focusing on effective implementation strategies in non-urban settings.

## REFERENCES

[1] Mensah GA, Roth GA, Sampson UKA, Moran AE, Feigin VL, Forouzanfar MH, et al.

Mortality from cardiovascular diseases in sub-Saharan Africa, 1990-2013: A systematic analysis of data from the Global Burden of Disease Study 2013. Cardiovasc J Afr 2015;26. https://doi.org/10.5830/CVJA-2015-036.

[2] Etyang AO, Scott JAG. Medical causes of admissions to hospital among adults in Africa: a systematic review. Glob Health Action 2013;6. https://doi.org/10.3402/GHA.V6I0.19090.
[3] Ogunmola OJ, Oladosu OY. Pattern and outcome of admissions in the medical wards of a tertiary health center in a rural community of Ekiti state, Nigeria. Ann Afr Med 2014;13. https://doi.org/10.4103/1596-3519.142291.
[4] Sadiq AM, Njau RE, Kilonzo KG. Disease patterns and clinical outcomes of medical admissions at a tertiary hospital in Northern Tanzania: A retrospective observational study. Health Sci Rep 2023;6. https://doi.org/10.1002/hsr2.983.
[5] Adoukonou T, Né Kossi O, Fotso Mefo P, Agbé Tou M, Magne J, Gbaguidi G, et al.

Stroke case fatality in sub-Saharan Africa: Systematic review and meta-analysis. Int J Stroke 2021;16:902–16. https://doi.org/10.1177/1747493021990945.

[6] Yao H, Ekou A, Niamkey T, Sandra ;, Gan H, Kouamé I, et al. SYSTEMATIC REVIEW AND META-ANALYSIS Acute Coronary Syndromes in Sub-Saharan Africa: A 10-Year Systematic Review. J Am Heart Assoc J Am Heart Assoc 2022;11:21107–21107. https://doi.org/10.1161/JAHA.120.021107.
[7] Dokainish H, Teo K, Zhu J, Roy A, Alhabib KF, Elsayed A, et al. Articles Global mortality variations in patients with heart failure: results from the International Congestive

Heart Failure (INTER-CHF) prospective cohort study. The Lancet 2017;5. https://doi.org/10.1016/S2214-109X(17)30196-1.

[8] Moran A, Forouzanfar M, Sampson U, Chugh S, Feigin V, Mensah G. The Epidemiology of Cardiovascular Diseases in Sub-Saharan Africa: The Global Burden of Diseases, Injuries and Risk Factors 2010 Study. Prog Cardiovasc Dis 2013;56:234–234. https://doi.org/10.1016/J.PCAD.2013.09.019.
[9] Fomonyuy Yuyun M, Sliwa K, Pascal Kengne A, Olga Mocumbi A, Bukhman G. Cardiovascular Diseases in Sub-Saharan Africa Compared to High-Income Countries: An Epidemiological Perspective. Glob Heart 2020;15:15–15. https://doi.org/10.5334/gh.403.
[10] Adeniji FIP, Obembe TA. Cardiovascular Disease and Its Implication for Higher Catastrophic Health Expenditures Among Households in Sub-Saharan Africa. J Health Econ Outcomes Res 2023;10:59–59. https://doi.org/10.36469/001C.70252.
[11] Watkins DA, Msemburi WT, Pickersgill SJ, Kawakatsu Y, Gheorghe A, Dain K, et al. NCD Countdown 2030: efficient pathways and strategic investments to accelerate progress towards the Sustainable Development Goal target 3.4 in low-income and middle- income countries. The Lancet 2022;399:1266–78. https://doi.org/10.1016/S0140- 6736(21)02347-3.
[12] 2023 ESC Guidelines for the management of acute coronary syndromes n.d. https://[www.escardio.org/Guidelines/Clinical-Practice-Guidelines/Acute-Coronary-](http://www.escardio.org/Guidelines/Clinical-Practice-Guidelines/Acute-Coronary-) Syndromes-ACS-Guidelines, https://[www.escardio.org/Guidelines/Clinical-Practice-](http://www.escardio.org/Guidelines/Clinical-Practice-) Guidelines/Acute-Coronary-Syndromes-ACS-Guidelines (accessed July 2, 2024).
[13] Perel P, Avezum A, Huffman M, Pais P, Rodgers A, Vedanthan R, et al. Reducing Premature Cardiovascular Morbidity and Mortality in People With Atherosclerotic

Vascular Disease The World Heart Federation Roadmap for Secondary Prevention of Cardiovascular Disease. Glob Heart 2015;10. https://doi.org/10.1016/j.gheart.2015.04.003.

[14] Hertz JT, Sakita FM, Kweka GL, Tarimo TG, Goli S, Prattipati S, et al. One-Year Outcomes and Factors Associated With Mortality Following Acute Myocardial Infarction in Northern Tanzania. Circ Cardiovasc Qual Outcomes 2022;15. https://doi.org/10.1161/CIRCOUTCOMES.121.008528.
[15] Yusuf S, Islam S, Chow CK, Rangarajan S, Dagenais G, Diaz R, et al. Use of secondary prevention drugs for cardiovascular disease in the community in high-income, middle- income, and low-income countries (the PURE Study): A prospective epidemiological survey. The Lancet 2011;378. https://doi.org/10.1016/S0140-6736(11)61215-4.
[16] Yoo SGK, Chung GS, Bahendeka SK, Sibai AM, Damasceno A, Farzadfar F, et al. Aspirin for Secondary Prevention of Cardiovascular Disease in 51 Low-, Middle-, and High- Income Countries. JAMA 2023;330. https://doi.org/10.1001/jama.2023.12905.
[17] Proctor EK, Powell BJ, McMillen JC. Implementation strategies: recommendations for specifying and reporting. Implement Sci 2013;8:139. https://doi.org/10.1186/1748- 5908-8-139.
[18] Powell BJ, Waltz TJ, Chinman MJ, Damschroder LJ, Smith JL, Matthieu MM, et al. A refined compilation of implementation strategies: results from the Expert Recommendations for Implementing Change (ERIC) project. Implement Sci 2015;10:21. https://doi.org/10.1186/s13012-015-0209-1.
[19] Aminde LN, Takah NF, Zapata-Diomedi B, Veerman JL. Primary and secondary prevention interventions for cardiovascular disease in low-income and middle-income

countries: a systematic review of economic evaluations. Cost Eff Resour Alloc CE 2018;16:22. https://doi.org/10.1186/s12962-018-0108-9.

[20] Page MJ, McKenzie JE, Bossuyt PM, Boutron I, Hoffmann TC, Mulrow CD, et al. The PRISMA 2020 statement: an updated guideline for reporting systematic reviews. BMJ 2021:n71. https://doi.org/10.1136/bmj.n71.
[21] Ouzzani M, Hammady H, Fedorowicz Z, Elmagarmid A. Rayyan-a web and mobile app for systematic reviews. Syst Rev 2016;5. https://doi.org/10.1186/s13643-016-0384-4.
[22] Kennedy CE, Fonner VA, Armstrong KA, Denison JA, Yeh PT, O’Reilly KR, et al. The Evidence Project risk of bias tool: Assessing study rigor for both randomized and non- randomized intervention studies. Syst Rev 2019;8. https://doi.org/10.1186/s13643- 018-0925-0.
[23] Mebazaa A, Davison B, Chioncel O, Cohen-Solal A, Diaz R, Filippatos G, et al. Safety, tolerability and efficacy of up-titration of guideline-directed medical therapies for acute heart failure (STRONG-HF): a multinational, open-label, randomised, trial. The Lancet 2022;400:1938–52. https://doi.org/10.1016/S0140-6736(22)02076-1.
[24] van Rooy L, Coopoo Y. Physical activity and lifestyle modification in the reduction of cardiovascular disease risk in coronary artery bypass graft patients Physical demands of rugby union View project MPhil Sport Science View project. Afr J Phys Act Health Sci 2017.
[25] De Villiers L, Zimba Kalula S, Burch VC. Does multidisciplinary stroke care improve outcome in a secondary-level hospital in South Africa? Int J Stroke 2009;4:89–93.
[26] Joughin HM, Digenio AG, Daly L, Kgare E. Physiological Benefits of a prolonged moderate intensity endurance training programme in patients with coronary artery disease. S Afr Med J 1999;89:545–9.
[27] Morris R, Digenio A, Padayachee GN, Kinnear B. The effect of a 6-month cardiac rehabilitation programme on serum lipoproteins and apoproteins Al and B and lipoprotein a. S Afr Med J 1993;83:315–8.
[28] Digenio A.G, Cantor A, Noakes T.D, Cloete L., Mavunda D., Esser J.D. Is severe left ventricular dysfunction a contraindication to participation in an exercise rehabilitation programme? S Afr Med J 1996.
[29] Owolabi MO, Gebregziabher M, Akinyemi RO, Akinyemi JO, Akpa O, Olaniyan O, et al.

Randomized Trial of an Intervention to Improve Blood Pressure Control in Stroke Survivors. Circ Cardiovasc Qual Outcomes 2019;12. https://doi.org/10.1161/CIRCOUTCOMES.119.005904.

[30] Awotidebe TO, Adedoyin RA, Balogun MO, Adebayo RA, Adeyeye VO, I. Oke K, et al.

Effects of Cardiac Rehabilitation Exercise Protocols on Physical Function in Patients with Chronic Heart Failure: An Experience from a Resource Constraint Nation. Int J Clin Med 2016;07:547–57. https://doi.org/10.4236/ijcm.2016.78060.

[31] Ajiboye OA, Anigbogu CN, Ajuluchukwu J. Therapeutic Effects of Exercise Training On Selected Cardio-Pulmonary Parameters and Body Composition of Nigerians with Chronic Heart Failure (A Preliminary Study) Endothelial Nitric Oxide Synthase Expression in Hypertension View project. 2013.
[32] Ajiboye OA, Anigbogu CN, Ajuluchukwu JN, Jaja SI. Exercise training improves functional walking capacity and activity level of Nigerians with chronic biventricular heart failure. Hong Kong Physiother J 2015;33:42–9. https://doi.org/10.1016/j.hkpj.2014.11.002.
[33] Sarfo FS, Treiber F, Gebregziabher M, Adamu S, Nichols M, Singh A, et al. Phone-based intervention for blood pressure control among Ghanaian stroke survivors: A pilot

randomized controlled trial. Int J Stroke 2019;14:630–8. https://doi.org/10.1177/1747493018816423.

[34] Anane C, Ohene-Buabeng K, Sarpong K, Owusu I. The Impact of a Comprehensive Medication Counseling and Education on Rehospitalization and Mortality of Advanced Heart Failure Patients in Ghana. J Cardiovasc Dis Diagn 2013;01. https://doi.org/10.4172/2329-9517.1000123.
[35] Wondesen A, Berha AB, Woldu M, Mekonnen D, Engidawork E. Impact of medication therapy management interventions on drug therapy problems, medication adherence and treatment satisfaction among ambulatory heart failure patients at Tikur Anbessa Specialised Hospital, Addis Ababa, Ethiopia: a one-group pre-post quasi-experimental study. BMJ Open 2022;12. https://doi.org/10.1136/bmjopen-2021-054913.
[36] Dessie G, Burrowes S, Mulugeta H, Haile D, Negess A, Jara D, et al. Effect of a self-care educational intervention to improve self-care adherence among patients with chronic heart failure: a clustered randomized controlled trial in Northwest Ethiopia. BMC Cardiovasc Disord 2021;21. https://doi.org/10.1186/s12872-021-02170-8.
[37] Nakibuuka J, Sajatovic M, Nankabirwa J, Ssendikadiwa C, Kalema N, Kwizera A, et al.

Effect of a 72 hour stroke care bundle on early outcomes after acute stroke: A non randomised controlled study. PLoS ONE 2016;11. https://doi.org/10.1371/journal.pone.0154333.

[38] Ahmed KO, Taj Eldin I, Yousif M, Albarraq AA, Yousef BA, Ahmed N, et al. Clinical Pharmacist’s Intervention to Improve Medication Titration for Heart Failure: First Experience from Sudan. Integr Pharm Res Pract 2021;Volume 10:135–43. https://doi.org/10.2147/iprp.s341621.
[39] Eberly LA, Rusingiza E, Park PH, Ngoga G, Dusabeyezu S, Mutabazi F, et al. 10-Year Heart Failure Outcomes From Nurse-Driven Clinics in Rural Sub-Saharan Africa. J Am Coll Cardiol 2019;73:977–80. https://doi.org/10.1016/j.jacc.2018.12.028.
[40] Ngeno GTK, Barasa F, Kamano J, Kwobah E, Wambui C, Binanay C, et al. Feasibility of Cardiac Rehabilitation Models in Kenya. Ann Glob Health 2022;88. https://doi.org/10.5334/aogh.3392.
[41] Kpadonou T, Fiogbé E, Datié A, Alagnidé E, Niama Nata D, Houngbedji G, et al.

Preliminary Results of a Cardiac Rehabilitation Program in Patients with Compensated Heart Diseases in Sub-Saharan Africa (Benin): A Pilot Study. Int J Phys Med Rehabil 2013;04. https://doi.org/10.4172/2329-9096.1000160.

[42] Walli-Attaei M, Joseph P, Johansson I, Sliwa K, Lonn E, Maggioni AP, et al.

Characteristics, management, and outcomes in women and men with congestive heart failure in 40 countries at different economic levels: an analysis from the Global Congestive Heart Failure (G-CHF) registry. Lancet Glob Health 2024;0. https://doi.org/10.1016/S2214-109X(23)00557-0.

[43] The World Bank. World Bank Open Data. World Bank Open Data 2022. https://data.worldbank.org (accessed January 27, 2024).
[44] G-CHF Investigators, Joseph P, Roy A, Lonn E, Störk S, Floras J, et al. Global Variations in Heart Failure Etiology, Management, and Outcomes. JAMA 2023;329:1650–61. https://doi.org/10.1001/jama.2023.5942.
[45] Kruk ME, Gage AD, Arsenault C, Jordan K, Leslie HH, Roder-DeWan S, et al. High-quality health systems in the Sustainable Development Goals era: time for a revolution. Lancet Glob Health 2018;6:e1196–252. https://doi.org/10.1016/S2214-109X(18)30386-3.
[46] Carlson S, Duber HC, Achan J, Ikilezi G, Mokdad AH, Stergachis A, et al. Capacity for diagnosis and treatment of heart failure in sub-Saharan Africa. Heart 2017;103:1874–9. https://doi.org/10.1136/heartjnl-2016-310913.
[47] Marcus ME, Manne-Goehler J, Theilmann M, Farzadfar F, Moghaddam SS, Keykhaei M, et al. Use of statins for the prevention of cardiovascular disease in 41 low-income and middle-income countries: a cross-sectional study of nationally representative, individual-level data. Lancet Glob Health 2022;10:e369. https://doi.org/10.1016/S2214-109X(21)00551-9.
[48] NIHR Open Data. NIHR Open Data n.d. https://nihr.opendatasoft.com/explore/?sort=modified (accessed June 6, 2024).

Supplementary material-Search criteria

GLOBAL HEALTH SEARCH

| **Query** | **Results from 8 Jan 2024** |  |
| --- | --- | --- |
| 1 | Patient Care Management.mp. | 17 |
| 2 | Comprehensive Health Care.mp. | 232 |
| 3 | Nursing Process.mp. | 90 |
| 4 | 'Nursing Assessment'.mp. | 43 |
| 5 | Patient Care Planning.mp. or planning.sh. or health care.sh. | 172,507 |
| 6 | Case Management.mp. | 3,521 |
| 7 | delivery of health care.mp. or primary health care.sh. | 25,782 |
| 8 | Delivery of Health Care, Integrated.mp. or health services.sh. | 125,672 |
| 9 | 'Managed Care Programs'.mp. | 7 |
| 10 | Disease Management.mp. | 3,736 |
| 11 | 'Patient Care Team'.mp. or physicians.sh. or Community care.sh. or nursing.sh. or general practitioners.sh. or patient care.sh. | 60,100 |
| 12 | 'Primary Health Care'.mp. or health care workers.sh. or health care.sh. or primary health care.sh. | 199,675 |
| 13 | Reminder Systems.mp. | 173 |
| 14 | Guideline Adherence.mp. | 384 |
| 15 | Home Care Services.mp. or medical services.sh. or health services.sh. | 130,409 |
| 16 | Home Nursing.mp. | 175 |
| 17 | 'Nursing Services'.mp. or nursing.sh. or nurses.sh. or medical services.sh. | 32,203 |
| 18 | 'Professional Role'.mp. or health care workers.sh. or medical auxiliaries.sh. | 56,549 |

| 19 | Community Health Services.mp. or community health services/ | 6,260 |
| --- | --- | --- |
| 20 | Medical Records/ or Medical Records Systems, Computerized.mp. [mp=abstract, title, original title, heading words, cabicodes words] | 5,584 |
| 21 | 'Patient Compliance'.mp. or patient compliance.sh. | 7,846 |
| 22 | Life Style.mp. or food intake.sh. or exercise.sh. or lifestyle.sh. or physical activity.sh. | 158,611 |
| 23 | Health Promotion.mp. or health promotion/ | 39,466 |
| 24 | Health Services Administration.mp. or health services.sh. | 125,707 |
| 25 | Education, Medical, Continuing.mp. or continuing education.sh. or medical education.sh. or education.sh. | 65,286 |
| 26 | Marketing of Health Services.mp. | 5 |
| 27 | Patient Participation.mp. or patient compliance.sh. or participation.sh. | 13,483 |
| 28 | Quality of Health Care.mp. or "quality of care".sh. | 12,551 |
| 29 | Quality Assurance, Health Care.mp. | 1 |
| 30 | Exercise/ or Physical Fitness.mp. [mp=abstract, title, original title, heading words, cabicodes words] | 46,497 |
| 31 | Smoking Cessation.mp. or smoking cessation/ | 11,879 |
| 32 | Diet/ or Fat-restricted/ or Carbohydrate-Restricted.mp. [mp=abstract, title, original title, heading words, cabicodes words] | 74,545 |
| 33 | (Alcohol Drinking prevention or control).mp. [mp=abstract, title, original title, heading words, cabicodes words] | 1,128,244 |
| 34 | Health Education.mp. or health education/ | 35,450 |
| 35 | Community Health Planning.mp. or community health.sh. or planning.sh. | 28,881 |
| 36 | Communication/ or Communication Barriers/ or Information Dissemination/ or Interdisciplinary Communication.mp. [mp=abstract, title, original title, heading words, cabicodes words] | 11,097 |
| 37 | Nurse Clinicians.mp. | 11 |

| 38 | Nurse Practitioners.mp. or nurses.sh. | 14,089 |
| --- | --- | --- |
| 39 | Risk Reduction Behavior.mp. or risk reduction.sh. | 22,200 |
| 40 | Pamphlets.mp. | 1,036 |
| 41 | Health Behavior.mp. or behaviour.sh. or health behaviour.sh. | 107,612 |
| 42 | Health Knowledge, Attitudes, Practice.mp. or attitudes.sh. or health education.sh. | 104,341 |
| 43 | Secondary Prevention.mp. or prevention.sh. | 34,745 |
| 44 | Preventive Health Services.mp. or preventive medicine.sh. | 6,412 |
| 45 | (manag$ adj3 care).tw. | 6,218 |
| 46 | (management adj3 program$).tw. | 5,345 |
| 47 | (case adj3 manag$).tw. | 5,078 |
| 48 | (patient adj3 management).tw. | 4,470 |
| 49 | (home adj3 intervention$).tw. | 1,004 |
| 50 | (home adj visit$).tw. | 3,568 |
| 51 | (discharg$ adj3 program$).tw. | 161 |
| 52 | (practice adj guideline$).tw. | 3,472 |
| 53 | (discharg$ adj3 plan$).tw. | 709 |
| 54 | (comprehensive adj3 care).tw. | 2,539 |
| 55 | (treatment adj3 plan$).tw. | 26,407 |
| 56 | (nurse$ adj3 led).tw. | 564 |
| 57 | (disease adj management).tw. | 3,736 |
| 58 | multidisciplin$.tw. | 13,055 |
| 59 | secondary prevention clinic$.tw. | 6 |
| 60 | reminder$.tw. | 3,651 |

| 61 | recall$.tw. | 23,082 |
| --- | --- | --- |
| 62 | (nurse adj3 clinic$).tw. | 413 |
| 63 | (secondary prevention adj3 intervention$).tw. | 107 |
| 64 | (secondary prevention adj3 program$).tw. | 148 |
| 65 | Appointments.mp. and Schedules/ [mp=abstract, title, original title, heading words, cabicodes words] | 0 |
| 66 | appointment$.tw. | 5,829 |
| 67 | (outreach adj nurs$).tw. | 11 |
| 68 | (outreach adj visit$).tw. | 84 |
| 69 | (lifestyle adj3 intervention$).tw. | 4,691 |
| 70 | (nurs$ adj intervention$).tw. | 649 |
| 71 | (education$ adj program$).tw. | 16,143 |
| 72 | (physical adj (activit$ or exercise$)).tw. | 80,579 |
| 73 | (exercise adj3 (train$ or intervention$ or program$ or activit$ or regim$)).tw. | 11,370 |
| 74 | fitness.tw. | 21,123 |
| 75 | (risk factor$ adj3 (modif$ or reduc$ or manage$ or monitor$ or self manage$)).tw. | 9,485 |
| 76 | (guideline directed medical therap* or GDMT).tw. | 25 |
| 77 | (guideline directed medical therap* or GDMT).mp. | 25 |
| 78 | (medic* adj3 uptit*).mp. | 3 |
| 79 | "Africa South of the Sahara".mp. or "Africa South of Sahara"/ | 268,733 |
| 80 | ("Africa South of the Sahara" or sub-Saharan Africa or subSaharan Africa).ti,ab. | 20,602 |
| 81 | Central Africa.tw. | 26,214 |
| 82 | Eastern Africa.tw. | 760 |

| 83 | Southern Africa.tw. | 64,686 |
| --- | --- | --- |
| 84 | Western Africa.tw. | 608 |
| 85 | Angola.mp. or Angola/ | 2,018 |
| 86 | Angola.ti,ab. | 1,805 |
| 87 | Cameroon.mp. or Cameroon/ | 9,645 |
| 88 | (Cameroon or Kamerun or Cameroun).ti,ab. | 8,046 |
| 89 | Cape Verde.mp. or Cape Verde/ | 447 |
| 90 | (Cape Verde or Cabo Verde).ti,ab. | 436 |
| 91 | Comoros.mp. or Comoros/ | 465 |
| 92 | (Comoros or Glorioso Islands or Mayotte).ti,ab. | 566 |
| 93 | Congo.mp. or Congo Basin/ or Congo/ or Congo River/ or Congo Democratic Republic/ | 15,348 |
| 94 | (Congo not ((Democratic Republic adj3 Congo) or congo red or crimean-congo)).ti,ab. | 5,333 |
| 95 | Cote d'Ivoire.mp. or Cote d'Ivoire/ | 5,763 |
| 96 | (Cote d'Ivoire or Cote dIvoire or Ivory Coast).ti,ab. | 5,333 |
| 97 | Eswatini.mp. or Eswatini/ | 1,160 |
| 98 | (eSwatini or Swaziland).ti,ab. | 1,109 |
| 99 | Ghana.mp. or Ghana/ | 14,960 |
| 100 | (Ghana or Gold Coast).ti,ab. | 13,445 |
| 101 | Kenya.mp. or Kenya/ | 23,690 |
| 102 | (Kenya or East Africa Protectorate).ti,ab. | 20,550 |
| 103 | Lesotho.mp. or Lesotho/ | 946 |
| 104 | (Lesotho or Basutoland).ti,ab. | 821 |
| 105 | Mauritania.mp. or Mauritania/ | 812 |

| 106 | Mauritania.ti,ab. | 709 |
| --- | --- | --- |
| 107 | Nigeria.mp. or Nigeria/ | 44,957 |
| 108 | Nigeria.ti,ab. | 37,515 |
| 109 | (Sao Tome adj2 Principe).ti,ab. | 161 |
| 110 | Senegal/ or Senegal.mp. | 8,277 |
| 111 | Senegal.ti,ab. | 6,447 |
| 112 | Sudan.mp. or Southern Sudan/ or South Sudan/ or Sudan/ | 10,460 |
| 113 | (Sudan not South Sudan).ti,ab. | 8,316 |
| 114 | Zambia.mp. or Zambia/ | 7,027 |
| 115 | (Zambia or Northern Rhodesia).ti,ab. | 6,029 |
| 116 | Zimbabwe.mp. or Zimbabwe/ | 8,182 |
| 117 | (Zimbabwe or Southern Rhodesia).ti,ab. | 5,851 |
| 118 | Botswana.mp. or Botswana/ | 2,626 |
| 119 | (Botswana or Bechuanaland or Kalahari).ti,ab. | 2,466 |
| 120 | Equatorial Guinea.mp. or Equatorial Guinea/ | 611 |
| 121 | (Equatorial Guinea or Spanish Guinea).ti,ab. | 514 |
| 122 | Gabon.mp. or Gabon/ | 2,225 |
| 123 | (Gabon or Gabonese Republic).ti,ab. | 1,899 |
| 124 | Mauritius.mp. or Mauritius/ | 1,217 |
| 125 | (Mauritius or Agalega Islands).ti,ab. | 1,072 |
| 126 | Namibia.mp. or Namibia/ | 1,571 |
| 127 | (Namibia or German South West Africa).ti,ab. | 1,215 |
| 128 | South Africa.mp. or South Africa/ | 42,987 |

| 129 | (South Africa or Cape Colony or British Bechuanaland or Boer Republics or Zululand or Transvaal or Natalia Republic or Orange Free State).ti,ab. | 30,860 |
| --- | --- | --- |
| 130 | Benin.mp. or Benin/ | 4,722 |
| 131 | (Benin or Dahomey).ti,ab. | 4,359 |
| 132 | Burkina Faso.mp. or Burkina Faso/ | 6,441 |
| 133 | (Burkina Faso or Burkina Fasso or Upper Volta).ti,ab. | 6,002 |
| 134 | Burundi.mp. or Burundi/ | 1,113 |
| 135 | (Burundi or Ruanda-Urundi).ti,ab. | 1,206 |
| 136 | Central African Republic/ | 1,260 |
| 137 | (Central African Republic or Ubangi-Shari).ti,ab. | 1,267 |
| 138 | Chad/ | 1,549 |
| 139 | Chad.ti,ab. | 1,580 |
| 140 | Democratic Republic of the Congo/ | 8,556 |
| 141 | (((Democratic Republic or DR) adj2 Congo) or Congo-Kinshasa or Belgian Congo or Zaire or Congo Free State).ti,ab. | 6,904 |
| 142 | Eritrea/ | 833 |
| 143 | Eritrea.ti,ab. | 773 |
| 144 | Ethiopia/ | 21,743 |
| 145 | (Ethiopia or Abyssinia).ti,ab. | 20,603 |
| 146 | Gambia/ | 3,410 |
| 147 | Gambia.ti,ab. | 2,939 |
| 148 | Guinea/ | 1,660 |
| 149 | (Guinea not (New Guinea or Guinea Pig* or Guinea Fowl or Guinea-Bissau or Portuguese Guinea or Equatorial Guinea)).ti,ab. | 3,422 |

| 150 | Guinea-Bissau/ | 1,158 |
| --- | --- | --- |
| 151 | (Guinea-Bissau or Portuguese Guinea).ti,ab. | 1,128 |
| 152 | (Guinea-Bissau or Portuguese Guinea).ti,ab. | 1,128 |
| 153 | Liberia/ | 2,027 |
| 154 | Liberia.ti,ab. | 2,036 |
| 155 | Madagascar/ | 4,433 |
| 156 | (Madagascar or Malagasy Republic).ti,ab. | 4,386 |
| 157 | Malawi/ | 8,386 |
| 158 | (Malawi or Nyasaland).ti,ab. | 7,715 |
| 159 | Mali/ | 3,914 |
| 160 | Mali.ti,ab. | 3,766 |
| 161 | Mozambique/ | 4,143 |
| 162 | (Mozambique or Mocambique or Portuguese East Africa).ti,ab. | 4,027 |
| 163 | Niger/ | 1,723 |
| 164 | (Niger not (Aspergillus or Peptococcus or Schizothorax or Cruciferae or Gobius or Lasius or Agelastes or Melanosuchus or radish or Parastromateus or Orius or Apergillus or Parastromateus or Stomoxys)).ti,ab. | 4,237 |
| 165 | Rwanda/ | 3,308 |
| 166 | (Rwanda or Ruanda).ti,ab. | 3,188 |
| 167 | Sierra Leone/ | 2,874 |
| 168 | (Sierra Leone or Salone).ti,ab. | 2,820 |
| 169 | Somalia/ | 2,212 |
| 170 | (Somalia or Somaliland).ti,ab. | 1,893 |

| 171 | South Sudan/ | 634 |
| --- | --- | --- |
| 172 | South Sudan.ti,ab. | 740 |
| 173 | Tanzania/ | 16,791 |
| 174 | (Tanzania or Tanganyika or Zanzibar).ti,ab. | 15,920 |
| 175 | Togo/ | 2,087 |
| 176 | (Togo or Togolese Republic or Togoland).ti,ab. | 1,905 |
| 177 | Uganda/ | 17,524 |
| 178 | Uganda.ti,ab. | 16,518 |
| 179 | Seychelles/ | 421 |
| 180 | Seychelles.ti,ab. | 422 |
| 181 | or/79-180 [ALL SUB-SAHARAN AFRICA COUNTRIES] | 289,036 |
| 182 | cardiovascular diseases.sh. | 69,616 |
| 183 | cardio*.tw. | 153,011 |
| 184 | cardia*.tw. | 52,067 |
| 185 | heart*.tw. | 144,945 |
| 186 | coronary*.tw. | 71,200 |
| 187 | angina*.tw. | 3,961 |
| 188 | ventric*.tw. | 18,566 |
| 189 | myocard*.tw. | 39,180 |
| 190 | pericard*.tw. | 4,066 |
| 191 | isch?em*.tw. | 30,174 |
| 192 | emboli*.tw. | 6,301 |
| 193 | arrhythmi*.tw. | 6,141 |

| 194 | thrombo*.tw. | 39,918 |
| --- | --- | --- |
| 195 | atrial fibrillat*.tw. | 3,616 |
| 196 | tachycardi*.tw. | 3,627 |
| 197 | endocardi*.tw. | 9,475 |
| 198 | (sick adj sinus).tw. | 59 |
| 199 | 'Stroke'/ | 16,845 |
| 200 | (stroke or stokes).tw. | 28,183 |
| 201 | cerebrovasc*.tw. | 12,041 |
| 202 | cerebral vascular.tw. | 467 |
| 203 | apoplexy.tw. | 196 |
| 204 | (brain adj2 accident*).tw. | 12 |
| 205 | ((brain* or cerebral or lacunar) adj2 infarct*).tw. | 3,239 |
| 206 | 'Hyperlipidemias'.mp. | 115 |
| 207 | hyperlipid*.tw. | 11,326 |
| 208 | hyperlip?emia*.tw. | 12,406 |
| 209 | hypercholesterol*.tw. | 13,098 |
| 210 | hypercholester?emia*.tw. | 204 |
| 211 | hyperlipoprotein?emia*.tw. | 1,165 |
| 212 | hypertriglycerid?emia*.tw. | 5,912 |
| 213 | 'Arteriosclerosis'.mp. | 22,257 |
| 214 | cholesterol/ or 'Cholesterol'.mp. | 109,109 |
| 215 | cholesterol.tw. | 109,109 |
| 216 | or/182-215 | 426,629 |

| 217 | or/1-78 | 1,775,836 |
| --- | --- | --- |
| 218 | 181 and 216 and 217 | 5,310 |

EMBASE SEARCH

| **Query** | **Results from 8 Jan 2024** |  |
| --- | --- | --- |
| 1 | Patient Care Management/ | 343,543 |
| 2 | Comprehensive Health Care/ | 136,567 |
| 3 | Nursing Process/ | 7,181 |
| 4 | 'nursing assessment'/ | 27,582 |
| 5 | Patient Care Planning/ | 31,171 |
| 6 | Case Management/ | 13,849 |
| 7 | delivery of health care/ | 177,915 |
| 8 | Delivery of Health Care, Integrated/ | 12,206 |
| 9 | Managed Care Programs'/ | 149,774 |
| 10 | Disease Management/ | 25,166 |
| 11 | Patient Care Team'/ | 376 |
| 12 | Primary Health Care'/ | 80,832 |
| 13 | Reminder Systems/ | 2,930 |
| 14 | Guideline Adherence/ | 18,628 |
| 15 | Home Care Services/ | 62,959 |
| 16 | Home Nursing/ | 71,590 |
| 17 | 'Nursing Services'/ | 232,814 |
| 18 | 'Professional Role'/ | 37,080 |
| 19 | Community Health Services/ | 58,630 |
| 20 | Medical Records/ or Medical Records Systems, Computerized/ | 195,311 |
| 21 | Patient Compliance'/ | 155,803 |

| 22 | Life Style/ | 127,087 |
| --- | --- | --- |
| 23 | Health Promotion/ | 113,237 |
| 24 | Health Services Administration/ | 182,986 |
| 25 | Education, Medical, Continuing/ | 254,245 |
| 26 | Marketing of Health Services/ | 121,705 |
| 27 | Patient Participation/ | 36,663 |
| 28 | Quality of Health Care/ | 237,273 |
| 29 | Quality Assurance, Health Care/ | 224,539 |
| 30 | Exercise/ or Physical Fitness/ | 408,815 |
| 31 | Smoking Cessation/ | 70,405 |
| 32 | Diet/ or Fat-restricted/ or Carbohydrate-Restricted/ | 316,655 |
| 33 | Alcohol Drinking/pc [Prevention & Control] | 1,060 |
| 34 | Health Education/ | 117,433 |
| 35 | Community Health Planning/ | 115,717 |
| 36 | Communication/ or Communication Barriers/ or Information Dissemination/ or Interdisciplinary Communication/ | 194,877 |
| 37 | Nurse Clinicians/ | 2,887 |
| 38 | Nurse Practitioners/ | 25,667 |
| 39 | Risk Reduction Behavior/ | 126,495 |
| 40 | Pamphlets/ | 195,947 |
| 41 | Health Behavior/ | 81,616 |
| 42 | Health Knowledge, Attitudes, Practice/ | 112,236 |
| 43 | Secondary Prevention/ | 36,003 |

| 44 | Preventive Health Services/ | 32,621 |
| --- | --- | --- |
| 45 | (manag$ adj3 care).ab,ti,kw. | 81,117 |
| 46 | (management adj3 program$).ab,ti,kw. | 33,111 |
| 47 | (case adj3 manag$).ab,ti,kw. | 37,262 |
| 48 | (patient adj3 management).ab,ti,kw. | 75,293 |
| 49 | (home adj3 intervention$).ab,ti,kw. | 5,874 |
| 50 | (home adj visit$).ab,ti,kw. | 14,800 |
| 51 | (discharg$ adj3 program$).ab,ti,kw. | 2,376 |
| 52 | (practice adj guideline$).ab,ti,kw. | 47,610 |
| 53 | (discharg$ adj3 plan$).ab,ti,kw. | 10,009 |
| 54 | (comprehensive adj3 care).ab,ti,kw. | 20,384 |
| 55 | (treatment adj3 plan$).ab,ti,kw. | 151,290 |
| 56 | (nurse$ adj3 led).ab,ti,kw. | 10,146 |
| 57 | (disease adj management).ab,ti,kw. | 31,367 |
| 58 | multidisciplin$.ab,ti,kw. | 201,059 |
| 59 | secondary prevention clinic$.ab,ti,kw. | 122 |
| 60 | reminder$.ab,ti,kw. | 26,523 |
| 61 | recall$.ab,ti,kw. | 123,395 |
| 62 | (nurse adj3 clinic$).ab,ti,kw. | 12,232 |
| 63 | (secondary prevention adj3 intervention$).ab,ti,kw. | 810 |
| 64 | (secondary prevention adj3 program$).ab,ti,kw. | 1,194 |
| 65 | Appointments.mp. and Schedules/ [mp=title, abstract, heading word, drug trade name, original title, device manufacturer, drug manufacturer, device trade name, keyword | 0 |

|  | heading word, floating subheading word, candidate term word] |  |
| --- | --- | --- |
| 66 | appointment$.ab,ti,kw. | 62,130 |
| 67 | (outreach adj nurs$).ab,ti,kw. | 215 |
| 68 | (outreach adj visit$).ab,ti,kw. | 445 |
| 69 | (lifestyle adj3 intervention$).ab,ti,kw. | 17,833 |
| 70 | (nurs$ adj intervention$).ab,ti,kw. | 10,866 |
| 71 | (education$ adj program$).ab,ti,kw. | 71,087 |
| 72 | (physical adj (activit$ or exercise$)).tw. | 233,523 |
| 73 | (exercise adj3 (train$ or intervention$ or program$ or activit$ or regim$)).ab,ti,kw. | 88,505 |
| 74 | fitness.ab,ti,kw. | 114,914 |
| 75 | (risk factor$ adj3 (modif$ or reduc$ or manage$ or monitor$ or self manage$)).ab,ti,kw. | 50,279 |
| 76 | (guideline directed medical therap* or GDMT).ab,ti,kw. | 2,530 |
| 77 | (guideline directed medical therap* or GDMT).mp. | 2,582 |
| 78 | (medic* adj3 uptit*).mp. | 80 |
| 79 | Africa South of the Sahara'/ | 18,412 |
| 80 | ("Africa South of the Sahara" or sub-Saharan Africa or subSaharan Africa).ab,ti. | 35,346 |
| 81 | Central Africa.ab,ti,kw. | 4,629 |
| 82 | Eastern Africa.ab,ti,kw. | 1,522 |
| 83 | Southern Africa.ab,ti,kw. | 6,175 |
| 84 | Western Africa.ab,ti,kw. | 1,153 |
| 85 | Angola/ | 1,896 |
| 86 | Angola.ab,ti. | 2,067 |
| 87 | Cameroon/ | 9,277 |

| 88 | (Cameroon or Kamerun or Cameroun).ab,ti. | 10,436 |
| --- | --- | --- |
| 89 | Cape Verde/ | 554 |
| 90 | (Cape Verde or Cabo Verde).ab,ti. | 832 |
| 91 | Comoros/ | 443 |
| 92 | (Comoros or Glorioso Islands or Mayotte).ab,ti. | 775 |
| 93 | Congo/ | 4,794 |
| 94 | (Congo not ((Democratic Republic adj3 Congo) or congo red or crimean-congo)).ab,ti. | 4,028 |
| 95 | Cote d'Ivoire/ | 3,998 |
| 96 | (Cote d'Ivoire or Cote dIvoire or Ivory Coast).ab,ti. | 5,433 |
| 97 | Eswatini/ | 357 |
| 98 | (eSwatini or Swaziland).ab,ti. | 1,413 |
| 99 | Ghana/ | 16,865 |
| 100 | (Ghana or Gold Coast).ab,ti. | 18,393 |
| 101 | Kenya/ | 27,636 |
| 102 | (Kenya or East Africa Protectorate).ab,ti. | 27,697 |
| 103 | Lesotho/ | 1,038 |
| 104 | (Lesotho or Basutoland).ab,ti. | 1,129 |
| 105 | Mauritania/ | 827 |
| 106 | Mauritania.ab,ti. | 866 |
| 107 | Nigeria/ | 48,843 |
| 108 | Nigeria.ab,ti. | 47,349 |
| 109 | (Sao Tome adj2 Principe).ab,ti. | 224 |
| 110 | Senegal/ | 8,238 |

| 111 | Senegal.ab,ti. | 8,259 |
| --- | --- | --- |
| 112 | Sudan/ | 8,795 |
| 113 | (Sudan not South Sudan).ab,ti. | 11,937 |
| 114 | Zambia/ | 8,321 |
| 115 | (Zambia or Northern Rhodesia).ab,ti. | 8,211 |
| 116 | Zimbabwe/ | 8,532 |
| 117 | (Zimbabwe or Southern Rhodesia).ab,ti. | 7,580 |
| 118 | Botswana/ | 3,498 |
| 119 | (Botswana or Bechuanaland or Kalahari).ab,ti. | 3,915 |
| 120 | Equatorial Guinea/ | 600 |
| 121 | (Equatorial Guinea or Spanish Guinea).ab,ti. | 700 |
| 122 | Gabon/ | 2,085 |
| 123 | (Gabon or Gabonese Republic).ab,ti. | 2,354 |
| 124 | Mauritius/ | 1,122 |
| 125 | (Mauritius or Agalega Islands).ab,ti. | 1,301 |
| 126 | Namibia/ | 2,136 |
| 127 | (Namibia or German South West Africa).ab,ti. | 2,280 |
| 128 | South Africa/ | 64,436 |
| 129 | (South Africa or Cape Colony or British Bechuanaland or Boer Republics or Zululand or Transvaal or Natalia Republic or Orange Free State).ti,ab. | 54,478 |
| 130 | Benin/ | 3,324 |
| 131 | (Benin or Dahomey).ab,ti. | 5,794 |
| 132 | Burkina Faso/ | 5,679 |

| 133 | (Burkina Faso or Burkina Fasso or Upper Volta).ab,ti. | 6,497 |
| --- | --- | --- |
| 134 | Burundi/ | 1,139 |
| 135 | (Burundi or Ruanda-Urundi).ab,ti. | 1,282 |
| 136 | Central African Republic/ | 1,109 |
| 137 | (Central African Republic or Ubangi-Shari).ab,ti. | 1,325 |
| 138 | Chad/ | 1,186 |
| 139 | Chad.ab,ti. | 1,972 |
| 140 | Democratic Republic of the Congo/ | 5,344 |
| 141 | (((Democratic Republic or DR) adj2 Congo) or Congo-Kinshasa or Belgian Congo or Zaire or Congo Free State).ab,ti. | 6,568 |
| 142 | Eritrea/ | 762 |
| 143 | Eritrea.ti,ab. | 886 |
| 144 | Ethiopia/ | 28,934 |
| 145 | (Ethiopia or Abyssinia).ti,ab. | 29,466 |
| 146 | Gambia/ | 3,412 |
| 147 | Gambia.ti,ab. | 3,203 |
| 148 | Guinea/ | 3,373 |
| 149 | (Guinea not (New Guinea or Guinea Pig* or Guinea Fowl or Guinea-Bissau or Portuguese Guinea or Equatorial Guinea)).ab,ti. | 3,993 |
| 150 | Guinea-Bissau/ | 1,283 |
| 151 | (Guinea-Bissau or Portuguese Guinea).ab,ti. | 1,345 |
| 152 | Liberia/ | 2,235 |
| 153 | Liberia.ti,ab. | 2,284 |

| 154 | Madagascar/ | 5,563 |
| --- | --- | --- |
| 155 | (Madagascar or Malagasy Republic).ab,ti. | 6,499 |
| 156 | Malawi/ | 10,045 |
| 157 | (Malawi or Nyasaland).ab,ti. | 10,764 |
| 158 | Mali/ | 4,368 |
| 159 | Mali.ti,ab. | 5,482 |
| 160 | Mozambique/ | 5,043 |
| 161 | (Mozambique or Mocambique or Portuguese East Africa).ab,ti. | 5,515 |
| 162 | Niger/ | 2,996 |
| 163 | (Niger not (Aspergillus or Peptococcus or Schizothorax or Cruciferae or Gobius or Lasius or Agelastes or Melanosuchus or radish or Parastromateus or Orius or Apergillus or Parastromateus or Stomoxys)).ti,ab. | 5,239 |
| 164 | Rwanda/ | 5,089 |
| 165 | (Rwanda or Ruanda).ab,ti. | 5,175 |
| 166 | Sierra Leone/ | 3,191 |
| 167 | (Sierra Leone or Salone).ab,ti. | 3,428 |
| 168 | Somalia/ | 2,577 |
| 169 | (Somalia or Somaliland).ab,ti. | 2,256 |
| 170 | South Sudan/ | 562 |
| 171 | South Sudan.ab,ti. | 958 |
| 172 | Tanzania/ | 20,287 |
| 173 | (Tanzania or Tanganyika or Zanzibar).ab,ti. | 20,999 |
| 174 | Togo/ | 1,865 |

| 175 | (Togo or Togolese Republic or Togoland).ab,ti. | 2,139 |
| --- | --- | --- |
| 176 | Uganda/ | 24,333 |
| 177 | Uganda.ab,ti. | 23,892 |
| 178 | Seychelles/ | 584 |
| 179 | Seychelles.ab,ti. | 930 |
| 180 | or/79-179 [ALL SUB-SAHARAN AFRICA COUNTRIES] | 413,514 |
| 181 | Cardiovascular Diseases'/ | 124,215 |
| 182 | cardio*.ab,ti,kw. | 1,526,046 |
| 183 | cardia*.ab,ti,kw. | 1,154,573 |
| 184 | heart*.ab,ti,kw. | 1,534,626 |
| 185 | coronary*.ab,ti,kw. | 697,481 |
| 186 | angina*.ab,ti,kw. | 92,422 |
| 187 | ventric*.ab,ti,kw. | 738,078 |
| 188 | myocard*.ab,ti,kw. | 679,750 |
| 189 | pericard*.ab,ti,kw. | 78,551 |
| 190 | isch?em*.ab,ti,kw. | 702,018 |
| 191 | emboli*.ab,ti,kw. | 242,201 |
| 192 | arrhythmi*.ab,ti,kw. | 180,615 |
| 193 | thrombo*.ab,ti,kw. | 692,395 |
| 194 | atrial fibrillat*.ab,ti,kw. | 173,332 |
| 195 | tachycardi*.ab,ti,kw. | 120,418 |
| 196 | endocardi*.ab,ti,kw. | 84,480 |
| 197 | (sick adj sinus).ab,ti,kw. | 3,876 |

| 198 | Stroke'/ | 212,103 |
| --- | --- | --- |
| 199 | (stroke or stokes).ab,ti,kw,tw. | 532,949 |
| 200 | cerebrovasc*.ab,ti,kw. | 111,371 |
| 201 | cerebral vascular.ab,ti,kw. | 10,883 |
| 202 | apoplexy.ab,ti,kw. | 4,936 |
| 203 | (brain adj2 accident*).ab,ti,kw. | 297 |
| 204 | ((brain* or cerebral or lacunar) adj2 infarct*).ab,ti,kw. | 48,182 |
| 205 | Hyperlipidemias'/ | 50,247 |
| 206 | hyperlipid*.ab,ti,kw. | 65,392 |
| 207 | hyperlip?emia*.ab,ti,kw. | 4,419 |
| 208 | hypercholesterol*.ab,ti,kw. | 60,706 |
| 209 | hypercholester?emia*.ab,ti,kw. | 1,345 |
| 210 | hyperlipoprotein?emia*.ab,ti,kw. | 6,262 |
| 211 | hypertriglycerid?emia*.ab,ti,kw. | 23,832 |
| 212 | Arteriosclerosis'/ | 41,359 |
| 213 | Cholesterol'/ | 261,452 |
| 214 | cholesterol.ab,ti,kw. | 397,027 |
| 215 | or/1-78 | 4,553,662 |
| 216 | or/181-214 | 5,364,254 |
| 217 | 180 and 215 and 216 | 5,464 |

MEDLINE SEARCH

| **Query** | **Results from 8 Jan 2024** |  |
| --- | --- | --- |
| 1 | Patient Care Management/ | 4,747 |
| 2 | Comprehensive Health Care/ | 6,807 |
| 3 | Nursing Process/ | 7,433 |
| 4 | exp Nursing Assessment/ | 33,070 |
| 5 | Patient Care Planning/ | 39,558 |
| 6 | Case Management/ | 10,684 |
| 7 | delivery of Health care/ | 120,366 |
| 8 | Delivery of Health Care, Integrated/ | 14,388 |
| 9 | exp Managed Care Programs/ | 40,338 |
| 10 | Disease Management/ | 43,062 |
| 11 | exp Patient Care Team/ | 73,083 |
| 12 | exp Primary Health Care/ | 194,547 |
| 13 | Reminder Systems/ | 3,811 |
| 14 | Guideline Adherence/ | 35,178 |
| 15 | Home Care Services/ | 36,621 |
| 16 | Home Nursing/ | 8,668 |
| 17 | exp Nursing Services/ | 52,474 |
| 18 | exp Professional Role/ | 89,508 |
| 19 | Community Health Services/ | 33,234 |
| 20 | Medical Records/ or Medical Records Systems, Computerized/ | 85,043 |

| 21 | exp Patient Compliance/ | 86,188 |
| --- | --- | --- |
| 22 | Life Style/ | 64,427 |
| 23 | Health Promotion/ | 82,135 |
| 24 | Health Services Administration/ | 4,555 |
| 25 | Education, Medical, Continuing/ | 25,624 |
| 26 | Marketing of Health Services/ | 14,841 |
| 27 | Patient Participation/ | 29,701 |
| 28 | Quality of Health Care/ | 77,811 |
| 29 | Quality Assurance, Health care/ | 56,932 |
| 30 | Exercise/ or Physical Fitness/ | 166,832 |
| 31 | Smoking Cessation/ | 33,117 |
| 32 | Diet/ or Fat-restricted/ or Carbohydrate-Restricted/ | 189,578 |
| 33 | Alcohol Drinking/pc [Prevention & Control] | 4,995 |
| 34 | Health Education/ | 64,004 |
| 35 | Community Health Planning/ | 5,247 |
| 36 | Communication/ or Communication Barriers/ or Information Dissemination/ | 127,364 |
| 37 | Nurse Clinicians/ | 8,561 |
| 38 | Nurse Practitioners/ | 19,012 |
| 39 | Risk Reduction Behavior/ | 14,322 |
| 40 | Pamphlets/ | 4,141 |
| 41 | Health Behavior/ | 57,043 |
| 42 | Health Knowledge, Attitudes, Practice/ | 127,618 |
| 43 | Secondary Prevention/ | 22,783 |

| 44 | Preventive Health Services/ | 14,501 |
| --- | --- | --- |
| 45 | manag$adj3 care.tw. | 0 |
| 46 | (management adj3 program$).tw. | 23,711 |
| 47 | (case adj3 manag$).tw. | 25,177 |
| 48 | (patient adj3 management).tw. | 48,196 |
| 49 | (home adj3 intervention$).tw. | 4,475 |
| 50 | (home adj visit$).tw. | 11,069 |
| 51 | (discharg$ adj3 program$).tw. | 1,384 |
| 52 | (practice adj guideline$).tw. | 33,801 |
| 53 | (discharg$ adj3 plan$).tw. | 6,110 |
| 54 | (comprehensive adj3 care).tw. | 13,718 |
| 55 | (treatment adj3 plan$).tw. | 97,396 |
| 56 | (nurse$ adj3 led).tw. | 6,105 |
| 57 | (disease adj management).tw. | 21,859 |
| 58 | multidisciplin$.tw. | 122,824 |
| 59 | secondary prevention clinic$.tw. | 69 |
| 60 | reminder$.tw. | 16,818 |
| 61 | recall$.tw. | 90,685 |
| 62 | (nurse adj3 clinic$).tw. | 7,801 |
| 63 | (secondary prevention adj3 intervention$).tw. | 532 |
| 64 | (secondary prevention adj3 program$).tw. | 782 |

| 65 | Appointments.mp. and Schedules/ [mp=title, book title, abstract, original title, name of substance word, subject heading word, floating sub-heading word, keyword heading word, organism supplementary concept word, protocol supplementary concept word, rare disease supplementary concept word, unique identifier, synonyms, population supplementary concept word, anatomy supplementary concept word] | 9,888 |
| --- | --- | --- |
| 66 | appointment$.tw. | 34,030 |
| 67 | (outreach adj nurs$).tw. | 104 |
| 68 | (outreach adj visit$).tw. | 339 |
| 69 | (lifestyle adj3 intervention$).tw. | 12,497 |
| 70 | (nurs$ adj intervention$).tw. | 8,648 |
| 71 | (education$ adj program$).tw. | 51,849 |
| 72 | (physical adj (activit$ or exercise$)).tw. | 171,361 |
| 73 | (exercise adj3 (train$ or intervention$ or program$ or activit$ or regim$)).tw. | 62,989 |
| 74 | fitness.tw. | 97,531 |
| 75 | (risk factor$ adj3 (modif$ or reduc$ or manage$ or monitor$ or self manage$)).tw. | 33,546 |
| 76 | (guideline directed medical therap* or GDMT).tw. | 1,081 |
| 77 | (guideline directed medical therap* or GDMT).mp. | 1,210 |
| 78 | (medic* adj3 uptit*).mp. | 33 |
| 79 | exp "Africa South of the Sahara"/ | 264,476 |
| 80 | ("Africa South of the Sahara" or sub-Saharan Africa or subSaharan Africa).ti,ab. | 30,139 |
| 81 | Central Africa.tw. | 3,865 |
| 82 | Eastern Africa.tw. | 1,317 |
| 83 | Southern Africa.tw. | 5,428 |
| 84 | Western Africa.tw. | 1,070 |

| 85 | Angola/ | 1,158 |
| --- | --- | --- |
| 86 | Angola.ti,ab. | 1,779 |
| 87 | Cameroon/ | 6,649 |
| 88 | (Cameroon or Kamerun or Cameroun).ti,ab. | 8,838 |
| 89 | Cape Verde/ | 259 |
| 90 | (Cape Verde or Cabo Verde).ti,ab. | 790 |
| 91 | Comoros/ | 384 |
| 92 | (Comoros or Glorioso Islands or Mayotte).ti,ab. | 745 |
| 93 | Congo/ | 2,031 |
| 94 | (Congo not ((Democratic Republic adj3 Congo) or congo red or crimean-congo)).ti,ab. | 3,043 |
| 95 | Cote d'Ivoire/ | 3,614 |
| 96 | (Cote d'Ivoire or Cote dIvoire or Ivory Coast).ti,ab. | 4,594 |
| 97 | Eswatini/ | 783 |
| 98 | (eSwatini or Swaziland).ti,ab. | 1,207 |
| 99 | Ghana/ | 11,542 |
| 100 | (Ghana or Gold Coast).ti,ab. | 15,785 |
| 101 | Kenya/ | 19,930 |
| 102 | (Kenya or East Africa Protectorate).ti,ab. | 23,307 |
| 103 | Lesotho/ | 542 |
| 104 | (Lesotho or Basutoland).ti,ab. | 985 |
| 105 | Mauritania/ | 509 |
| 106 | Mauritania.ti,ab. | 764 |
| 107 | Nigeria/ | 34,729 |

| 108 | Nigeria.ti,ab. | 37,272 |
| --- | --- | --- |
| 109 | (Sao Tome adj2 Principe).ti,ab. | 204 |
| 110 | Senegal/ | 6,286 |
| 111 | Senegal.ti,ab. | 6,685 |
| 112 | Sudan/ | 5,231 |
| 113 | (Sudan not South Sudan).ti,ab. | 8,920 |
| 114 | Zambia/ | 5,631 |
| 115 | (Zambia or Northern Rhodesia).ti,ab. | 6,908 |
| 116 | Zimbabwe/ | 6,651 |
| 117 | (Zimbabwe or Southern Rhodesia).ti,ab. | 7,059 |
| 118 | Botswana/ | 2,234 |
| 119 | (Botswana or Bechuanaland or Kalahari).ti,ab. | 3,316 |
| 120 | Equatorial Guinea/ | 322 |
| 121 | (Equatorial Guinea or Spanish Guinea).ti,ab. | 523 |
| 122 | Gabon/ | 1,647 |
| 123 | (Gabon or Gabonese Republic).ti,ab. | 2,065 |
| 124 | Mauritius/ | 631 |
| 125 | (Mauritius or Agalega Islands).ti,ab. | 1,192 |
| 126 | Namibia/ | 1,331 |
| 127 | (Namibia or German South West Africa).ti,ab. | 2,086 |
| 128 | South Africa/ | 50,219 |
| 129 | (South Africa or Cape Colony or British Bechuanaland or Boer Republics or Zululand or Transvaal or Natalia Republic or Orange Free State).ti,ab. | 45,395 |

| 130 | Benin/ | 1,963 |
| --- | --- | --- |
| 131 | (Benin or Dahomey).ti,ab. | 4,288 |
| 132 | Burkina Faso/ | 4,054 |
| 133 | (Burkina Faso or Burkina Fasso or Upper Volta).ti,ab. | 5,451 |
| 134 | Burundi/ | 731 |
| 135 | (Burundi or Ruanda-Urundi).ti,ab. | 1,156 |
| 136 | Central African Republic/ | 850 |
| 137 | (Central African Republic or Ubangi-Shari).ti,ab. | 1,198 |
| 138 | Chad/ | 832 |
| 139 | Chad.ti,ab. | 1,538 |
| 140 | Democratic Republic of the Congo/ | 5,134 |
| 141 | (((Democratic Republic or DR) adj2 Congo) or Congo-Kinshasa or Belgian Congo or Zaire or Congo Free State).ti,ab. | 5,566 |
| 142 | Eritrea/ | 427 |
| 143 | Eritrea.ti,ab. | 725 |
| 144 | Ethiopia/ | 20,052 |
| 145 | (Ethiopia or Abyssinia).ti,ab. | 28,709 |
| 146 | Gambia/ | 2,736 |
| 147 | Gambia.ti,ab. | 2,788 |
| 148 | Guinea/ | 1,339 |
| 149 | (Guinea not (New Guinea or Guinea Pig* or Guinea Fowl or Guinea-Bissau or Portuguese Guinea or Equatorial Guinea)).ti,ab. | 3,350 |
| 150 | Guinea-Bissau/ | 1,040 |

| 151 | (Guinea-Bissau or Portuguese Guinea).ti,ab. | 1,195 |
| --- | --- | --- |
| 152 | Liberia/ | 1,415 |
| 153 | Liberia.ti,ab. | 1,952 |
| 154 | Madagascar/ | 3,951 |
| 155 | (Madagascar or Malagasy Republic).ti,ab. | 5,774 |
| 156 | Malawi/ | 6,853 |
| 157 | (Malawi or Nyasaland).ti,ab. | 9,227 |
| 158 | Mali/ | 2,742 |
| 159 | Mali.ti,ab. | 4,456 |
| 160 | Mozambique/ | 3,101 |
| 161 | (Mozambique or Mocambique or Portuguese East Africa).ti,ab. | 4,748 |
| 162 | Niger/ | 1,443 |
| 163 | (Niger not (Aspergillus or Peptococcus or Schizothorax or Cruciferae or Gobius or Lasius or Agelastes or Melanosuchus or radish or Parastromateus or Orius or Apergillus or Parastromateus or Stomoxys)).ti,ab. | 4,190 |
| 164 | Rwanda/ | 3,220 |
| 165 | (Rwanda or Ruanda).ti,ab. | 4,302 |
| 166 | Sierra Leone/ | 1,982 |
| 167 | (Sierra Leone or Salone).ti,ab. | 2,972 |
| 168 | Somalia/ | 1,930 |
| 169 | (Somalia or Somaliland).ti,ab. | 2,054 |
| 170 | South Sudan/ | 285 |
| 171 | South Sudan.ti,ab. | 824 |

| 172 | Tanzania/ | 14,170 |
| --- | --- | --- |
| 173 | (Tanzania or Tanganyika or Zanzibar).ti,ab. | 17,751 |
| 174 | Togo/ | 1,297 |
| 175 | (Togo or Togolese Republic or Togoland).ti,ab. | 1,847 |
| 176 | Uganda/ | 15,772 |
| 177 | Uganda.ti,ab. | 19,543 |
| 178 | Seychelles/ | 456 |
| 179 | Seychelles.ti,ab. | 905 |
| 180 | or/79-179 [ALL SUB-SAHARAN AFRICA COUNTRIES] | 362,146 |
| 181 | exp Cardiovascular Diseases/ | 2,756,349 |
| 182 | cardio*.tw. | 959,856 |
| 183 | cardia*.tw. | 726,042 |
| 184 | heart*.tw. | 983,247 |
| 185 | coronary*.tw. | 451,360 |
| 186 | angina*.tw. | 59,283 |
| 187 | ventric*.tw. | 466,771 |
| 188 | myocard*.tw. | 440,038 |
| 189 | pericard*.tw. | 49,107 |
| 190 | isch?em*.tw. | 451,130 |
| 191 | emboli*.tw. | 150,271 |
| 192 | arrhythmi*.tw. | 107,802 |
| 193 | thrombo*.tw. | 428,576 |
| 194 | atrial fibrillat*.tw. | 92,622 |

| 195 | tachycardi*.tw. | 69,202 |
| --- | --- | --- |
| 196 | endocardi*.tw. | 56,518 |
| 197 | (sick adj sinus).tw. | 2,524 |
| 198 | exp Stroke/ | 177,624 |
| 199 | (stroke or stokes).tw. | 329,127 |
| 200 | cerebrovasc*.tw. | 67,450 |
| 201 | cerebral vascular.tw. | 6,613 |
| 202 | apoplexy.tw. | 3,536 |
| 203 | (brain adj2 accident*).tw. | 186 |
| 204 | ((brain* or cerebral or lacunar) adj2 infarct*).tw. | 32,485 |
| 205 | exp Hyperlipidemias/ | 71,182 |
| 206 | hyperlipid*.tw. | 37,442 |
| 207 | hyperlip?emia*.tw. | 2,546 |
| 208 | hypercholesterol*.tw. | 39,360 |
| 209 | hypercholester?emia*.tw. | 782 |
| 210 | hyperlipoprotein?emia*.tw. | 4,375 |
| 211 | hypertriglycerid?emia*.tw. | 15,237 |
| 212 | exp Arteriosclerosis/ | 201,349 |
| 213 | exp Cholesterol/ | 175,606 |
| 214 | cholesterol.tw. | 278,652 |
| 215 | or/181-214 | 4,543,941 |
| 216 | or/1-78 | 2,421,993 |
| 217 | 180 and 215 and 216 | 3,655 |

Figure	[Click here to access/download;Figure;Fig 1.pdf](https://www2.cloud.editorialmanager.com/pgph/download.aspx?id=384557&guid=a6e7561d-d660-4bae-a13d-b41940a8db00&scheme=1)[<https://www2.cloud.editorialmanager.com/pgph/download.aspx?id=384557&guid=a6e7561d-d660-4bae-a13d-b41940a8db00&scheme=1>](https://www2.cloud.editorialmanager.com/pgph/download.aspx?id=384557&guid=a6e7561d-d660-4bae-a13d-b41940a8db00&scheme=1)

Figure	[Click here to access/download;Figure;Fig 2.pdf](https://www2.cloud.editorialmanager.com/pgph/download.aspx?id=384558&guid=f790e18a-d0c8-47c4-8965-f287a4aa5a30&scheme=1)[<https://www2.cloud.editorialmanager.com/pgph/download.aspx?id=384558&guid=f790e18a-d0c8-47c4-8965-f287a4aa5a30&scheme=1>](https://www2.cloud.editorialmanager.com/pgph/download.aspx?id=384558&guid=f790e18a-d0c8-47c4-8965-f287a4aa5a30&scheme=1)

Supporting Information

# [Click here to access/download](https://www2.cloud.editorialmanager.com/pgph/download.aspx?id=387199&guid=57397ccd-c003-432f-a018-62149993aa79&scheme=1)

[Supporting Information PRISMA_CVD SSA.docx](https://www2.cloud.editorialmanager.com/pgph/download.aspx?id=387199&guid=57397ccd-c003-432f-a018-62149993aa79&scheme=1)
