# Supplementary material for: Implementation strategies to improve outcomes in patients with established cardiovascular disease in sub-Saharan Africa: A systematic review
Source: PLOS Glob Public Health. 2025 May 5;5(5):e0004544. doi: 10.1371/journal.pgph.0004544 (PMC12052185; doi:10.1371/journal.pgph.0004544)
Supplement: Attachment — Submitted filename: response.docx [file pgph.0004544.s006.docx]

***Editors’ comments:***

**1. Your current Financial Disclosure states, “NIHR”. However, your funding information on the submission form indicates that you received funding from “National Institute for Health and Care Research” and Grant Recipient "Pablo Perel". Please indicate by return email the full and correct funding information for your study and confirm the order in which funding contributions should appear. Please be sure to indicate whether the funders played any role in the study design, data collection and analysis, decision to publish, or preparation of the manuscript.**

*The review was funded by the National Institute for Health and Care Research (NIHR). The grant number is GHR NIHR134544. The Principal investigators of the grant are Professor Pablo Perel and Dr Anthony Etyang.*

**2. We ask that a manuscript source file is provided at Revision. Please upload your manuscript file as a .doc, .docx, .rtf or .tex.**

*Thank you and done.*

**3. Please provide separate figure files in .tif or .eps format.**

**For more information about figure files please see our guidelines:**

[**https://journals.plos.org/globalpublichealth/s/figures**](https://url.uk.m.mimecastprotect.com/s/1PzbCzXkjig4g88u4f0C95ii1?domain=journals.plos.org) 
[**https://journals.plos.org/globalpublichealth/s/figures#loc-file-requirements**](https://url.uk.m.mimecastprotect.com/s/Vwq4CA1pkc6E6rrf8hoCGfBuh?domain=journals.plos.org)

*Thank you and done.*

**4. We notice that your supplementary "Supplementary material-Search criteria" are included in the manuscript file. Please remove them and upload them with the file type 'Supporting Information'. Please ensure that each Supporting Information file has a legend listed in the manuscript after the references list.**

*Thank you and done.*

**5. Please provide a complete Data Availability Statement in the submission form, ensuring you include all necessary access information or a reason for why you are unable to make your data freely accessible. If your research concerns only data provided within your submission, please write "All data are in the manuscript and/or supporting information files" as your Data Availability Statement.**

*Our research concerns only data provided within the submission as it represents a literature review. We have stated that ‘All data are in the manuscript and/or supporting information files’*

**6. Figure 2: please (a) provide a direct link to the base layer of the map (i.e., the country or region border shape) and ensure this is also included in the figure legend; and (b) provide a link to the terms of use / license information for the base layer image or shapefile. We cannot publish proprietary or copyrighted maps (e.g. Google Maps, Mapquest) and the terms of use for your map base layer must be compatible with our CC-BY 4.0 license.**

**Note: if you created the map in a software program like R or ArcGIS, please locate and indicate the source of the basemap shapefile onto which data has been plotted.**

**If your map was obtained from a copyrighted source please amend the figure so that the base map used is from an openly available source. Alternatively, please provide explicit written permission from the copyright holder granting you the right to publish the material under our CC-BY 4.0 license.**

**Please note that the following CC BY licenses are compatible with PLOS license: CC BY 4.0, CC BY 2.0 and CC BY 3.0, meanwhile such licenses as CC BY-ND 3.0 and others are not compatible due to additional restrictions.**

**If you are unsure whether you can use a map or not, please do reach out and we will be able to help you. The following websites are good examples of where you can source open access or public domain maps: 
* U.S. Geological Survey (USGS) - All maps are in the public domain. (**[**http://www.usgs.gov**](http://url.uk.m.mimecastprotect.com/s/y4rPCB6q0U686PPfNikC2lSow?domain=usgs.gov)**) 
* PlaniGlobe - All maps are published under a Creative Commons license so please cite “PlaniGlobe,** [**http://www.planiglobe.com**](http://url.uk.m.mimecastprotect.com/s/GXpmCD8vnCv3vjjcZsBCjxlF7?domain=planiglobe.com)**, CC BY 2.0” in the image credit after the caption. (**[**http://www.planiglobe.com/?lang=enl**](http://url.uk.m.mimecastprotect.com/s/iWWuCExwoH9p911hZtXC71dBz?domain=planiglobe.com)**) 
* Natural Earth - All maps are public domain. (**[**http://www.naturalearthdata.com/about/terms-of-use/**](http://url.uk.m.mimecastprotect.com/s/DliGCG80qCYAYWWh0uwCBsiiX?domain=naturalearthdata.com)**)**

*Thank you. The base layer of the maps were made using the QGIS software (*[*https://www.qgis.org/*](https://www.qgis.org/)*). The base layer used were those provided within QGIS software ecosystem.*

*We can confirm that licensing of the maps from QGIS fall under CC BY 3.0. The link to this information is here:* [*https://docs.qgis.org/2.18/en/docs/gentle_gis_introduction/gnu_free_documentation_license.html*](https://docs.qgis.org/2.18/en/docs/gentle_gis_introduction/gnu_free_documentation_license.html)

**7. Please review your reference list to ensure that it is complete and correct. If you have cited papers that have been retracted, please include the rationale for doing so in the manuscript text, or remove these references and replace them with relevant current references. Any changes to the reference list should be mentioned in the rebuttal letter that accompanies your revised manuscript. If you need to cite a retracted article, indicate the article’s retracted status in the References list and also include a citation and full reference for the retraction notice.**

*Thank you and done*

**Response to reviewers**

**Reviewer #1:**

**The main objective of the research article was to describe, synthesize and identify key gaps in the implementation strategies of evidence-based approaches that improves clinical outcomes for patients with CVD in SSA.

The strength of the manuscript is to provide helpful insights about gaps in implementation strategies that improve care outcomes of CVD patients in SSA. The research article describes extremely important health topic of interest, contributing to preventable mortality from CVDs creating substantial tall of already strained health systems of SSA countries. The article calls for increased investments, including in in interventions and research targeting care of patient with CVD to achieve SDGs.**

*We thank the reviewer for these insightful comments and for taking the time to review our manuscript.*

**The actionability of the manuscript could be improved by standardizing description of interventions and better systematizing implementation strategies by different domains. This will help to differentiate clinical interventions from various implementation strategies from one hand and identifying explicit gaps in implementation strategies from another to inform programmatic and learning agenda.**

*This is a very helpful suggestion. We have now taken this on board responding to the clear steer provided for table 1 (please see our point-by-point response below).*

**Specific recommendations are detailed below.
The lines of the manuscript are not numbered per PLOS LaTeX template. In the review, I will therefore highlight the feedback per sections without indicating line numbers.

Exclusion criteria
• It may be helpful to add primary prevention of CVD as an exclusion criterion, considering that study explicitly focuses on acute or established CVD. If this criterion was considered at a later stage (during expert consensus or consultation with 3rd reviewer or during the read of preselected 34 studies), please, state so.**

*We did exclude studies evaluating populations for primary prevention of cardiovascular disease. We have now addressed this (page 5, paragraph 2).*

**Table 1
• To better identify and systematize gap in implementation strategies, it would be helpful to 1st classify evidence-based interventions for management of patients with CVD beyond pharmacologic/non-pharmacologic.
• Similarly, implementation strategies could be further systematized by interventions targeting patient or families (demand side); improving point-of care service provision (point of care interventions) and broader, above site, health system strengthening interventions.**

*Thank you and please see our response below.*

**• When systematizing the interventions by various implementation strategies (e.g. task sharing, model of care), it would be helpful to refer to any specific systematization used for these purposes noting key domains used to systematize interventions. For example, the research article sites EPIC implementation strategy paper in the discussion, which provides compiled list of strategies to implement change in clinical settings (**[**https://implementationscience.biomedcentral.com/articles/10.1186/s13012-015-0209-1/tables/3**](https://url.uk.m.mimecastprotect.com/s/hka5CLJEyfjQjNNF1HNCyAsqB?domain=implementationscience.biomedcentral.com)**). There may be other, more relevant systematization of implementation strategies that could be used to better describe interventions. This will help to better differentiate clinical interventions from various implementation strategies from one hand and identifying gaps in implementation strategies from another.**

*Thank you. We have reviewed the EPIC implementation strategy paper alongside the systematic review published by the Cochrane EPOC Group (*[*https://www.ncbi.nlm.nih.gov/pmc/articles/PMC5621088/*](https://www.ncbi.nlm.nih.gov/pmc/articles/PMC5621088/)*). After discussion by the authors and given the particular relevance to low-resource setting, we have decided to use the framework of the Cochrane EPOC group. In summary,*

A) *We have made table 1 more concise and readable*
B) *We have created a new table 2 which lists the intervention type, strategy and strategy target (healthcare recipient, healthcare provider or the organisation)*

**• Suggest using referenced article (Proctor, E.K., Powell, B.J. & McMillen, J.C. Implementation strategies: recommendations for specifying and reporting. Implementation Sci 8, 139 (2013).** [**https://doi.org/10.1186/1748-5908-8-139**](https://url.uk.m.mimecastprotect.com/s/yXfbCM1GzcOzOxxuRIEC8on7M?domain=doi.org)**) to specify each implementation strategy, described in the table 1**

*Thank you and done.*

**Discussion**
**• Page 2 states that “only three studies [25,29,39] recruited patients from non-teaching or non-tertiary care settings. This finding has particular relevance to the region, given that nearly 6 out 10 people in SSA do not reside in urban regions.[43]” Another important reason for this could be limited availability of CVD care in lower-level health facilities in SSA.**

*Thank you and we have amended the discussion to reflect this (page 15, paragraph 1).*

**• The fact that studies covered only 10 out of over 50 SSA countries limit generalizability of the study findings to entire SSA countries and further demonstrate substantial evidence gap in CVD care implementation best practices in the region. I suggest to explicitly state these in relevant sections of the article.**

*Thank and a very important point that we have now highlighted (page 15, paragraph 1).*

**Conclusion
• Gaps in implementation strategies related to evaluating diagnostics could be partly due to limiting the systematic review to already diagnosed CVDs. Suggest removing “evaluating diagnostics” from the conclusion unless you have a strong justification.**

**• Suggest revising sentence” Particular gaps were highlighted in the acute care setting, specifically evaluating diagnostics and pharmacotherapeutic optimizations” as “Particular gaps were highlighted in the acute care setting, specifically related to acute coronary syndrome and implementation strategies targeting pharmacotherapeutic optimizations.”**

*Thank you and revised.*

**• As noted earlier, limited number of studies in non-tertiary and teaching hospital settings may be related to poor availability of CVD care in lower-level health facilities. I therefore suggest reviewing the sentence: “We also highlight a notable lack of studies focusing on effective implementation strategies in non-urban settings” as “We also highlight a notable lack of studies focusing on effective implementation strategies in primary care facilities and lower-level hospital settings”.**

*Thank you and revised.*

**Reviewer #2: PLOS Global Public Health
The authors have presented an insightful research with a concise and clear title. The research has a good contextual background with relevant research objectives. However, the authors need to pay attention to some of the highlighted observations in the manuscript presented.**

*We thank the reviewer for these insightful comments and for taking the time to review our manuscript.*

**There were numerous typographical errors, inconsistencies in writings and representations.**

*We apologise for the typographical errors. We have reviewed the manuscript carefully and addressed this.*

**The references were improperly cited with inconsistent referencing style. The tables appear cumbersome with some fragmented words.**

*We have now reviewed the references and addressed any inconsistencies.*

**While reading through the manuscript, there was a noticeable disconnect between the objectives, results and discussion. The page numbering was not serialized; the authors should carefully address all these concerns. (Check comments on the attachment).**

*We have improved the relevant sections as suggested by reviewer one and hope that the readability has improved. We have also addressed the refereeing issues and the typographical errors that the reviewer has kindly highlighted.*

**I commend the authors for this meta-analysis study which truly will bridge a gap in knowledge not only in Sub-Saharan Africa but worldwide.**

*Thank you.*

**Reviewer #3: This systematic review aims to synthesise and highlight critical gaps in the implementation of evidence-based cardiovascular disease (CVD) interventions in Sub-Saharan Africa. The manuscript has several strengths, including adherence to PRISMA guidelines, a comprehensive database search, clear eligibility criteria, and the use of the Evidence Project risk of bias tool. The review identifies significant gaps in the implementation of CVD interventions, particularly in acute care settings and for hospitalized patients.**

*We thank the reviewer for these insightful comments and for taking the time to review our manuscript.*

***While the authors are off to a good start, they should provide more details to enhance the methodological rigor of the review. Additionally, I recommend a thorough proofreading of the manuscript for editorial issues, including formatting the tables, enhancing clarity, and spelling out numbers below ten.***

*We have provided additional details on the methods including how we have classified the implementation strategies (see comments from Reviewer 1) and details on the exclusion criteria etc. We have also addressed the formatting and typographical issues encountered and apologise for this.*

**Methods:
1. The authors state that the study was reported in accordance with the PRISMA guidelines (Page 6, Data source and search strategy). However, the PRISMA checklist is not included in the manuscript or supplementary file.**

*Thank you. We have provided this***.**

**2. The authors should indicate if the review protocol was pre-registered in PROSPERO.**

*The review was registered on PROSPERO and the registration number detailed at the end of the manuscript. We have now quoted the registration number in the main body of the text.*

**3. The authors mention that the bibliography of key papers was searched (Page 6, Data source and search strategy), but it is unclear what these key papers are and how they were identified.**

*We found 6 papers through the bibliographic search. We have clarified this in the revised manuscript (page 8, paragraph 1) and amended the PRISMA flowchart (figure 1) accordingly.*

**4. The authors mention that they did not restrict their search by language (Page 6, Data source and search strategy). It would be helpful to clarify whether any of the screened studies were in languages other than the primary language of the review and how such studies were managed, including any translation processes or resources used to ensure accurate data extraction and analysis.**

*We did not find any studies that were not written in English. We have clarified this in the revised manuscript (page 8, paragraph 1).*

**5. The authors state that CVD was restricted to recurrent myocardial infarction, stroke, heart failure, and ischemic heart disease (Page 6, inclusion criteria), but it is unclear why other forms of CVD (such as peripheral artery disease) were excluded.**

*We concentrated on these three conditions as the top three cardiovascular pathologies contributing to disease burden in the region. We have stated this in the discussion and as a limitation of our review.*

**6. While the authors use the Evidence Project risk of bias tool (Page 7/8, evidence synthesis and risk of bias), it would be helpful to explain why this specific tool was chosen over others.**

*We specifically chose this risk of bias tool as it encompassed both randomised and non-randomised intervention studies. We have clarified this in the revised manuscript (page 17, paragraph 2),*

**7. Although the description of the risk of bias tool's criteria is clear, there is no information on how the scores were interpreted or what thresholds were used to categorise studies as having high or low risk of bias.**

*Thank you for raising this important issue. The risk of bias tool does not offer a specific threshold that differentiates high from low risk of bias. Instead, it aims to provides a numerical value to be interpreted relative to the other studies. The authors of the risk of bias tool state that the different items and domains do not assess an equal risk of bias and as such a threshold to define risk of bias has not been recommended.*
