# Supplementary material for: Implementation strategies to improve outcomes in patients with established cardiovascular disease in sub-Saharan Africa: A systematic review
Source: PLOS Glob Public Health. 2025 May 5;5(5):e0004544. doi: 10.1371/journal.pgph.0004544 (PMC12052185; doi:10.1371/journal.pgph.0004544)
Supplement: Attachment — Submitted filename: response_to_reviewer.docx [file pgph.0004544.s007.docx]

***Editors’ comments:***

**The reviewers are largely happy with the revision, but have requested a few further clarifications to the text, further discussion, and further proofreading. Could you please revise the manuscript to carefully address the concerns raised?**

Thank you and we have addressed the comments below, point-by-point.

***Reviewers’ comments:***

**1. On page 12, under 'Stroke', there is a comment that there was no difference in outcomes between the 'multidisciplinary stroke unit with the medical ward' and the 'stroke care bundle at a tertiary hospital'. It is not clear from this paragraph or Table 1 what was the key difference between these 2 implementation strategies. It would be useful to highlight if there were any key differences (even in the cost of these 2 strategies, for example).**

Thank you. The comment refers to the research paper by Nakibuuka et al. The comparison was made of implementing a formal acute stroke care pathway which included haemodynamic and glycaemic monitoring; brain imaging; and administration of pharmacotherapy. Funds were made available to deliver and implement the care pathway but differences in the costs of these two strategies were not detailed in the paper. Importantly, the acute care pathway was implemented for a period of 72 hours, with usual care from that point on. We have now detailed this point in the revised manuscript (page 12, paragraph 3). **2. While the conclusion understandably highlighted the many gaps, there may have been some studies from which useful intervention strategies could be identified (e.g. the ones highlighted in point 1 above) - and the authors could, perhaps, include a 'what next' or a section on 'recommendations' - e.g. identifying the more promising interventions to ascertain if they can be replicated in more rural settings, as you have mentioned that 60% of the SSA population live in a rural setting. This would make for a more robust paper that doesn't just identify issues but make sound recommendations to move this important issue forward.**

Thank you and a great suggestion. We have now included a recommendation section in the revised manuscript, just before the conclusions (page 17, paragraph 3). **3. A few typos still to be corrected - Pg 10 - '"health failure" instead of "heart failure". Pg 19 - para beginning with "Provision of..." - 2nd sentence has "across SSA" twice. Please check rest of document too.**

Thank you. We apologise for the typographical errors and have corrected these.
